# Mechanisms Underlying the Impact of Interleukin Family on Acute Kidney Injury: Pathogenesis, Progression, and Therapy

**DOI:** 10.34133/research.0738

**Published:** 2025-06-13

**Authors:** Yuwei Ji, Zhenkai Zhao, Yan Yang, Xiaochen Wang, Ruifeng Qiao, Xiang Yu, Xinyan Gong, Zhe Feng, Quan Hong

**Affiliations:** ^1^ Department of Nephrology, First Medical Center of Chinese PLA General Hospital, State Key Laboratory of Kidney Diseases, National Clinical Research Center for Kidney Diseases, Beijing Key Laboratory of Medical Devices and Integrated Traditional Chinese and Western Drug Development for Severe Kidney Diseases, Beijing Key Laboratory of Digital Intelligent TCM for the Prevention and Treatment of Pan-vascular Diseases, Key Disciplines of National Administration of Traditional Chinese Medicine (zyyzdxk-2023310), Beijing 100853, China.; ^2^ Department of Burns and Plastic Surgery, Fourth Medical Center of Chinese PLA General Hospital, Beijing 100048, China.; ^3^ Seventh Medical Center of Chinese PLA General Hospital, Beijing 100700, China.

## Abstract

Acute kidney injury (AKI) is a clinical syndrome with high mortality, and its pathogenesis involves complex inflammatory regulatory mechanisms. As core components of the cytokine network, interleukins (ILs) exert pleiotropic effects in the development of AKI, participating in processes such as inflammation, fibrosis, tissue damage repair, and remote organ injury. Moreover, ILs influence the progression of AKI by mediating the crosstalk among renal resident cells, immune cells, and fibroblasts. Pro-inflammatory ILs primarily accelerate the progression of AKI by recruiting neutrophils and inducing renal cell apoptosis, whereas anti-inflammatory ILs alleviate AKI by inhibiting the release of inflammatory cytokines and enhancing regulatory T cell function. Dual-function ILs may either promote disease progression or facilitate tissue repair depending on their cellular origin or the specific pathological stage. In terms of therapeutic strategies, monoclonal antibodies targeting ILs and their receptors, as well as advancements in extracellular vesicle technology, have shown promising potential. Future research should focus on elucidating the specific signaling networks of ILs and their intercellular interactions in order to promote precision medicine approaches for AKI and to block the transition from AKI to chronic kidney disease (CKD).

## Introduction

Acute kidney injury (AKI), a multifactorial pathological condition, affects approximately 10% to 15% of hospitalized patients and up to 50% in intensive care unit (ICU) settings, markedly elevating mortality risk [1]. AKI can be triggered by ischemia, toxins, and other factors that alter the immune microenvironment and induce inflammation, leading to cellular death [2]. Single-cell transcriptomics has revealed dynamic changes in immune cell populations during AKI, impacting injury outcomes [3]. Adaptive as well as innate immune responses play a role, with some immune cells exacerbating AKI, whereas others offer protection [4].

Inflammatory mediators are central to the origins of AKI, particularly those released by the damaged renal tissue or activated immune cell [5]. Tubular epithelial cell (TEC) death initiates a cascade of innate immune responses that release endogenous substances termed damage-associated molecular patterns (DAMPs), triggering pattern recognition receptor activation on resident cells or leukocyte recruitment [6]. This results in the release of proinflammatory cytokines and chemokines, attracting more inflammatory cells and creating a cycle that amplifies tissue damage [7]. Sterile inflammation from DAMPs is one of the earliest processes following injury that influences early adaptive immune responses, affecting both kidney damage and repair, as well as potential injuries to distant organs [6,8].

Interleukins (ILs), as key members of the cytokine family, are low-molecular-weight signaling proteins secreted by a variety of immune and nonimmune cells, playing crucial roles in orchestrating immune responses. Notably, during the pathogenesis of AKI, distinct ILs exert diverse biological effects through unique signaling pathways. For example, IL-1α, IL-1β, IL-12, IL-17A/C, and IL-18 [9,10] worsen AKI by enhancing inflammation and immune activation, whereas IL-2, IL-10, IL-13, IL-27, and IL-37 [11] have protective effects. Some ILs, including IL-4, IL-6, IL-9, IL-11, IL-22, and IL-33 [12–14], exhibit dual roles that vary according to the stage of AKI or experimental conditions. Understanding these mechanisms is vital for developing innovative therapies and improving outcomes in patients with AKI.

## Origin and Structure of the IL Family

The discovery of ILs began in the 1970s, during a period of groundbreaking advances in immunology. In 1979, at the Second International Lymphokine Symposium, IL-1 was formally named as the first systematically characterized member of the IL family [15]. As prototypical pleiotropic cytokines, ILs are produced not only by adaptive immune cells such as CD4^+^ T cells, B cells, monocytes/macrophages, and dendritic cells (DCs) but also by nonimmune cells including endothelial and epithelial cells [16]. Immune cells primarily produce various ILs, such as IL-1β, IL-2, and IL-10 [17–19], following activation by pathogen-associated molecular patterns (PAMPs) or cytokine signaling pathways [20]. A classical mechanism involves TLR-mediated nuclear factor κB (NF-κB) activation, which promotes cytokine transcription, along with inflammasome-dependent maturation and release of IL-1β and IL-18 via caspase-1 cleavage [21]. Local microenvironmental signals drive helper T cell (Th cell) differentiation and specific IL secretion through signal transducer and activator of transcription (STAT) signaling pathways. For example, IL-12/interferon-γ (IFN-γ) activates STAT4 to promote Th1 cell differentiation and secretion of IL-2 and IFN-γ [17], while IL-4 signals via STAT6 to induce Th2 cells to secrete IL-4, IL-5, and IL-13 [22–25]. DCs and monocytes/macrophages secrete IL-23, which drives Th17 cell activation and subsequent production of IL-17A/F, IL-6, and IL-22 [18]. Regulatory T cells (Tregs) depend on CD25-STAT5 signaling to capture IL-2 and secrete IL-10 [26]. Regulatory B cells (Bregs), upon stimulation by antigens, TLR ligands (e.g., CpG), or cytokines (e.g., IL-6 and IFN-α), activate the STAT3/IRF4 (interferon regulatory factor 4) axis to produce IL-10 and IL-35 [19]. Under the combined action of IFN-γ and signals from B cell receptor (BCR), TLR, and CD40, B cells can also generate IL-6 [27]. In monocytes/macrophages, the NLRP3 inflammasome mediates the cleavage of pro-IL-1β and pro-IL-18 into their mature forms via caspase-1 [28,29]. This process typically involves 2 steps: First, lipopolysaccharide (LPS) induces the expression of pro-IL-1β, pro-IL-18, and NLRP3 components through the TLR4/NF-κB pathway [30]; second, adenosine triphosphate (ATP) triggers potassium efflux via the P2X7 receptor, leading to NLRP3 inflammasome assembly, caspase-1 activation, and subsequent release of mature IL-1β and IL-18 [31]. Notably, LPS alone mainly stimulates macrophages to secrete IL-6 [29]. In addition to the canonical NLRP3 pathway, renal TECs possess a noncanonical inflammasome pathway mediated by caspase-11 in humans (caspase-4/5 in mice) [32,33]. DCs recognize pathogenic signals via TLRs, up-regulate major histocompatibility complex (MHC) and costimulatory molecules, and secrete IL-12, IL-1β, and IL-23, thereby promoting Th1 and Th17 cell differentiation [34]. Natural killer (NK) cells regulate IL-10 production through STAT phosphorylation in response to stimulation by IL-12, IL-15, IL-18, IL-21, and IL-27 [35]. Compared with immune cells, intrinsic renal cells—such as renal TECs and glomerular endothelial cells—are capable of producing multiple ILs, including IL-1 (e.g., IL-1β), IL-6, IL-8, and IL-33 [36–38], under conditions of ischemia, toxin exposure, or immune activation, primarily through NF-κB and TLR signaling pathways. The maturation and secretion of IL-1β in these cells are similarly dependent on NLRP3 inflammasome-mediated caspase-1 activation [39].

**Fig. 1.**
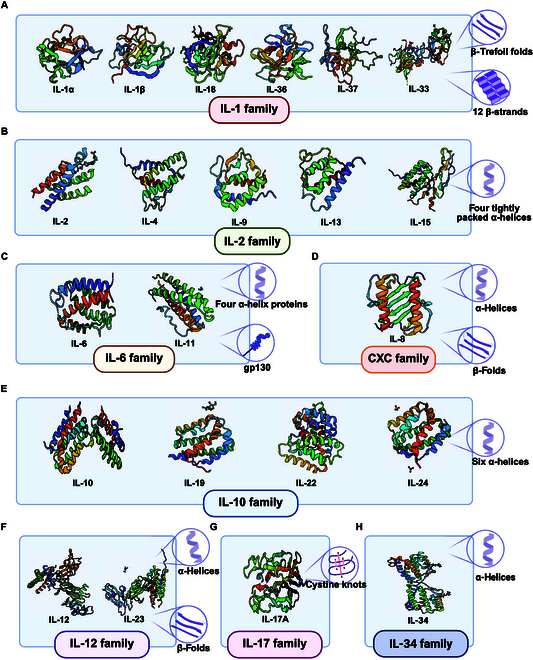
Gallery of IL proteins: their 3D structural features. This figure displays the 3D structures of proteins from 8 families: IL-1, IL-2, IL-6, CXC, IL-10, IL-12, IL-17, and IL-34. Common structural features within each family are highlighted in the circles on the right for easy reference. (A) IL-1 family signature structure: β-trefoil fold; 12 β-strands. (B) IL-2 family signature structure: 4 tightly packed α-helices. (C) IL-6 family signature structure: 4 α-helix proteins; gp130. (D) CXC family signature structure: α-helices; β-fold. (E) IL-10 family signature structure: 6 α-helices. (F) IL-12 family signature structure: α-helices; β-fold. (G) IL-17 family signature structure: cystine knot. (H) IL-34 family signature structure: α-helices. Figure created with BioRender.com.

There is some structural resemblance across the members of the IL family, despite their varied biological activities and capabilities. Most ILs are small polypeptides or proteins, exhibiting molecular weights between 15 and 30 kDa. Although amino acid sequences vary markedly among different ILs, members within the same family frequently exhibit conserved sequence regions. For instance, members of the IL-1 family possess a conserved β-trefoil fold and hydrophobic core composed of 12 β-strands (Fig. 1a) [40]. IL-2 family members feature a “4-helix bundle” structure formed by 4 tightly packed α-helices (Fig. 1B) [41]. Cytokines in the IL-6 family are also 4-helix proteins that commonly utilize the glycoprotein 130 (gp130) receptor subunit for signaling (Fig. 1C) [42]. The IL-10 family members are characterized by 6 α-helices, with 4 of these helices forming the canonical “4-helix bundle” structure commonly associated with cytokines (Fig. 1E) [43]. The IL-12 family is the only heterodimeric cytokine family that consists of an α-subunit and a β-subunit that associates noncovalently to form a functional heterodimer (Fig. 1F) [44]. Members of the IL-17 family have a distinctive structure characterized by 4 conserved cysteine residues in their protein sequences, which may form a specialized “cysteine knot” conformation (Fig. 1G) [45]. Furthermore, certain ILs exist as homodimers or heterodimers, with some forming higher-order multimeric structures. Overall, the structure of ILs is closely related to their function, and their distinct spatial conformations and domains endow them with the ability to carry out a variety of biological tasks inside the immune system.

## The Role of the IL Family in AKI

ILs are classified into distinct subgroups based on differences in receptor structure and functional properties, and they exert diverse regulatory roles in AKI (Table 1). ILs derived from immune cells versus intrinsic renal cells exhibit functionally distinct characteristics. Immune-derived ILs, such as IL-1β released by macrophages and IL-17A secreted by Th17 cells, predominantly drive early inflammatory responses by promoting neutrophil recruitment and complement activation, thereby exacerbating tubulointerstitial injury [46,47]. In contrast, ILs originating from intrinsic renal cells, such as IL-33 released by epithelial cells and IL-8 produced by endothelial cells, sustain injury signals and modulate the balance between tissue repair and fibrosis [48,49]. Notably, IL-10 exhibits functional heterogeneity depending on its cellular source. IL-10 secreted by Tregs exerts anti-inflammatory effects by suppressing excessive immune activation, whereas IL-10 produced by interstitial cells in diabetic kidneys may promote fibrosis through synergistic interactions with transforming growth factor-β (TGF-β) [50]. This functional divergence highlights the importance of considering the cellular origin of ILs when designing therapeutic strategies for AKI. Moreover, IL-34, specifically produced by intrinsic renal cells, promotes macrophage proliferation and monocyte infiltration [51,52], underscoring the unique role of resident renal cells in modulating the local microenvironment.

**Table 1.** Summary of the roles of interleukin families in AKI

Interleukin family	Cytokine	Receptor	Source	Mechanism of action	Type of effect	Reference
IL-1 family	IL-1α	IL-1R1, IL-1R2	Renal tubular epithelial cells, mesangial cells, macrophages, neutrophils, endothelial cells, fibroblasts	Exerts pro-inflammatory effects	Exacerbating	[9,206]
	IL-1β	IL-1R1, IL-1R2	Dendritic cells, macrophages, neutrophils	Enhances inflammation; damages glomerular barrier	Exacerbating	[207]
	IL-18	IL-18Rα, IL-18Rβ	Macrophages, renal tubular epithelial cells, intercalated cells, renal interstitial cells	Increases inflammation, promotes M2 macrophage shift to myofibroblasts, driving fibrosis and tissue remodeling	Exacerbating	[91,92]
	IL-33	ST2, IL-1RAcP	Endothelial cells, renal tubular epithelial cells, fibroblasts	(a) Promotes Th2 response and kidney protection; (b) enhances inflammation and fibrosis post-IRI	Dual functions	[14,173,179–183,185]
	IL-36	IL-36R/IL-1RAcP	Renal tubular epithelial cells, renal interstitial cells, neutrophils, macrophages, T lymphocytes, B lymphocytes, monocytes	Activates NF-κB and up-regulates IL-6 and TNF-α	Exacerbating	[99,100]
	IL-37	IL-18Rα, IL-1R8	Renal tubular epithelial cells, monocytes, macrophages	Alleviates hypoxia, restricts cytokine expression, improves monocyte infiltration and renal function	Mitigating	[67]
IL-2 family	IL-2	IL-2Rγ, IL-2Rα/IL-2Rα, IL-2Rβ/IL-2Rβ/IL-2Rγ	Activated T cells	Enhances Tregs, reduces ROS damage and apoptosis, improves renal function post-IRI	Mitigating	[109–118]
	IL-4	IL-4Rα/IL-13Rα1, IL-4Rα/γc	Th2 cells, macrophages, dendritic cells, B lymphocytes	(a) Promotes M2 macrophage polarization for tubular recovery, protects against albumin-induced TII; (b) accelerates fibrosis by promoting fibroblast activation in AKI models.	Dual functions	[148,150–152]
	IL-9	IL-9R (IL-9Rα/γc)	Podocytes, Th9 cells, ILC2s, Vδ2 T cells, mast cells	(a) Enhances resident cell secretion to promote inflammation; (b) protects kidney function and structure in cisplatin-induced AKI.	Dual functions	[186–188]
	IL-13	IL-4Rα/IL-13Rα1	Th2 cells	Promotes M2 polarization for AKI recovery; activates JAK-STAT to reduce apoptosis and promote regeneration	Mitigating	[143]
	IL-15	IL-15R (IL-15Rα/IL-15Rβ/γc)	Macrophages, dendritic cells, endothelial cells	Activation of the JAK/STAT and PI3K/Akt signaling pathways to prevent apoptosis and promote survival of renal cells	Mitigating	[133–136]
IL-6 family	IL-6	IL-6R/gp130	Renal tubular epithelial cells, podocytes, and mesangial cells are examples of kidney-resident cells	(a) IL-6 activates STAT3, prevents AKI, boosts IL-10; (b) MECP2 blocks IL-6/STAT3, protects kidneys; low IL-6 improves function, reduces neutrophils	Dual functions	[164–168]
	IL-11	IL-11R	Bone marrow stromal cells	(a) Boosts HIF-1α to induce SK1, reducing cell death and inflammation; (b) accelerates renal fibrosis in AKI models	Dual functions	[189,190]
IL-8 family	IL-8	CXCR1, CXCR2	Renal tubular epithelial cells, podocytes, monocytes, macrophages, T cells, vascular endothelial cells	Recruits neutrophils, mediates local inflammation	Exacerbating	[67–70]
IL-10 family	IL-10	IL-10Rα, IL-10Rβ	Th2 cells, Tregs, macrophages, dendritic cells	Activates JAK1/TYK2-STAT for anti-inflammation; inhibits immune cells, reduces factors, suppresses inflammation	Mitigating	[124–129]
	IL-19	IL-20R1/IL-20R2	Macrophages, dendritic cells, fibroblasts	Up-regulates TGF-β1, MCP-1, IL-19; activates pathways; promotes apoptosis; worsens inflammation; increases damage	Exacerbating	[102]
	IL-20	IL-20R1/IL-20R2, IL-22R1/IL-20R2	Renal tubular epithelial cells, macrophages, dendritic cells, neutrophils	Induces G0/G1 arrest, apoptosis; activates ERK1/2, boosts inflammation; promotes ROS/iNOS, renal fibrosis via TGF-β	Exacerbating	[103]
	IL-22	IL-22R1/IL-10R2	Proximal tubule epithelial cells, interstitial dendritic cells, macrophages	(a) Increases STAT3/Akt phosphorylation, up-regulates Bcl-2, down-regulates Bad; (b) activates DDR, promotes apoptosis	Dual functions	[13,171]
	IL-24	IL-20R1/IL-20R2,IL-22R1/IL-20R2	Renal tubular epithelial cells, infiltrating inflammatory cells	Induces apoptosis, enhances ER stress, up-regulates TGF-β1, PDGF-B, CTGF	Exacerbating	[94–96]
IL-12 family	IL-12	IL-12Rβ1/IL-12Rβ2	Dendritic cells, macrophages, B lymphocytes	Activates Th1, links immunities, promotes inflammation	Exacerbating	[75–77]
	IL-23	IL-23R/IL-12Rβ1	Dendritic cells, macrophages, B lymphocytes	Stimulates IL-17A, recruits neutrophils, boosts defense and autoimmunity	Exacerbating	[104–106]
	IL-27	IL-27Rα/gp130	Dendritic cells, macrophages	Increases STAT3 expression, promotes phosphorylation, prevents renal IRI	Mitigating	[144]
	IL-35	IL-12Rβ2/gp130	Tregs, dendritic cells, macrophages, B lymphocytes	Elevates IL-10, enhances Tregs, inhibits Teffs, suppresses inflammation	Mitigating	[145]
IL-17 family	IL-17A	IL-17RA/IL-17RC	Th17 cells, γδT cells, mast cells	Increases inflammation, induces neutrophil infiltration and apoptosis, promotes SA-AKI	Exacerbating	[83–87]
	IL-17C	IL-17RA/IL-17RE	Renal tubular epithelial cells	Recruits Th17 cells, worsens local inflammation	Exacerbating	[10,101]
	IL-17E	IL-17RA/IL-17RB	T lymphocytes, mast cells, eosinophils, basophils	Increases ILC2s/MPP2, improves renal function; activates M2, inhibits M1	Mitigating	[137–139,141,142]
IL-34 family	IL-34	CSF-1R	Renal tubular epithelial cells	Promotes macrophage damage, worsens ischemia-induced AKI	Exacerbating	[52,107]

Within the kidney, ILs do not act in isolation but instead mediate complex crosstalk among renal parenchymal cells, immune cells, and fibroblasts, collectively influencing the progression of inflammation, tissue repair, and fibrosis during AKI. TEC-derived factors such as TGF-β and IL-1β can induce endothelial–mesenchymal transition (EndMT), playing a crucial role in the development of renal fibrosis [53]. Additionally, under hypoxic conditions, IL-1β signaling via the IL-1 receptor (IL-1R) induces cell cycle arrest and senescence in renal TECs, promoting a pro-inflammatory and pro-fibrotic senescence-associated secretory phenotype (SASP), which further aggravates interstitial fibrosis [54]. Recent studies have revealed that injured TECs can also secrete IL-11, leading to fibroblast activation and extracellular matrix (ECM) deposition [55]. Conversely, IL-22, primarily secreted by immune cells, promotes the repair and regeneration of renal TECs, exerting anti-inflammatory and anti-fibrotic effects that contribute to the amelioration of AKI-induced tissue damage [56]. In a model of cardiorenal syndrome following AKI, blockade of IL-33 markedly attenuated myocardial hypertrophy and renal fibrosis, highlighting the therapeutic potential of targeting inter-organ communication pathways [57]. Collectively, these findings illustrate that ILs dynamically regulate the signaling networks among renal cells, immune cells, and fibroblasts, balancing pro-inflammatory responses with tissue repair processes, and thus represent promising therapeutic targets in AKI.

## ILs Exacerbating AKI Progression

**Fig. 2.**
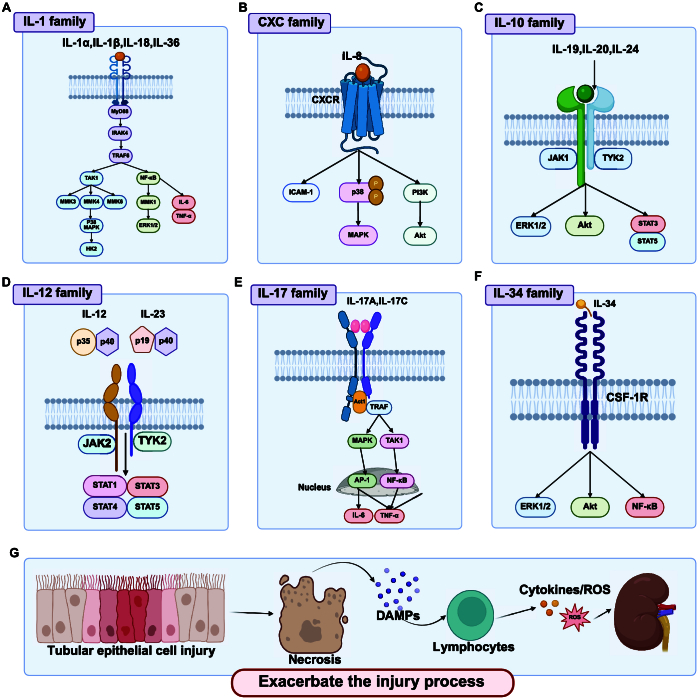
ILs exacerbating AKI progression. This figure illustrates the mechanisms by which various cytokine families facilitate the progression of AKI. (A) IL-1 family: Signaling pathways involving IL-1α, IL-1β, IL-18, and IL-36, highlighting their interactions with MyD88 and IRAK4, leading to activation of NF-κB and MAPK pathways. (B) CXC family: Role of IL-8 in AKI progression through its interaction with CXCR receptors, leading to activation of ICAM-1, p38 MAPK, and PI3K/Akt pathways. (C) IL-10 family: Signaling pathways of IL-19, IL-20, and IL-24, showing their interactions with JAK1/STAT3 and TYK2/STAT5 pathways. (D) IL-12 family: Signaling pathways of IL-12 and IL-23, focusing on the p35/p40 and p19/p40 subunits and their interactions with JAK2/STAT1 and TYK2/STAT3 pathways. (E) IL-17 family: Role of IL-17A and IL-17C in AKI progression, showing their interactions with TRAF, MAPK, TAK1, AP-1, and NF-κB pathways. (F) IL-34 family: Signaling pathway involving CSF-1R, ERK1/2, Akt, and NF-κB. (G) Overview of AKI progression: Overall process, including TEC injury, necrosis, DAMP release, lymphocyte activation, and the promotion of further injury through cytokines and ROS. Figure created with BioRender.com.

IL-1 is a key pro-inflammatory cytokine that participates in the pathogenesis of various kidney diseases by recruiting inflammatory responses [58]. IL-1α is constitutively present in keratinocytes and other epithelial cells (such as renal TECs), whereas macrophages, granulocytes, endothelial cells, fibroblasts, and mesangial cells express pro-IL-1α only upon activation [58]. Upon cell injury, IL-1α is released as an “alarmin”, activating neighboring cells and inducing endothelial cells to express adhesion molecules, thereby promoting leukocyte infiltration [59]. In contrast, IL-1β is mainly secreted by tissue-resident DCs, infiltrating macrophages, and neutrophils, while parenchymal cells produce only small amounts under pathological stimuli [58]. The production of IL-1β follows a classical pathway involving TLR/NF-κB-induced transcription of pro-IL-1β and inflammasome-mediated cleavage by caspase-1 into its mature form [28–31]. Mature IL-1α and IL-1β bind to IL-1R1, initiating downstream signaling cascades that play complex and diverse roles in AKI induced by different etiologies. In ischemia–reperfusion-induced AKI, IL-1 stimulates inflammatory cascades, leading to increased inflammatory cell infiltration and tissue damage. However, activation of IL-1R1 on CD11c^+^ myeloid cells promotes the expression of the anti-inflammatory factor IL-1R antagonist (IL-1Ra), limiting IL-1β-induced tubular cell injury in vitro and ameliorating AKI [60]. In nephrotoxic drug-induced AKI, IL-1α gene knockout (KO) substantially attenuates cisplatin-induced AKI [9]. In contrast, activation of IL-1R1 exacerbates kidney injury by promoting neutrophil infiltration and stimulating tumor necrosis factor (TNF) production by myeloid cells [61]. Interestingly, as the common receptor for both IL-1α and IL-1β, IL-1R1 exhibits cell type-dependent effects in AKI. Activation of IL-1R1 in renal TECs aggravates cell injury and metabolic reprogramming, promoting apoptosis and dysfunction [62]. Conversely, activation of IL-1R1 in renal endothelial cells improves aristolochic acid (AA)-induced AKI by restoring vascular endothelial growth factor A (VEGFA)-dependent endothelial cell viability and density [62]. In podocytes, IL-1R1 activation limits albuminuria and podocyte injury during nephrotoxic serum (NTS)-induced and doxorubicin-induced nephropathy through stimulation of the intracellular Akt signaling cascade [62]. Therefore, given the cell-specific effects of IL-1R signaling in different renal cell types during AKI, we cannot simply conclude that IL-1 solely promotes disease progression. Instead, the treatment of AKI may require precise targeting of IL-1 signaling from different cellular sources to achieve kidney-protective effects (Fig. 2A and Fig. S1).

IL-8, or chemokine ligand 8 (CXCL8), is an important proinflammatory chemokine that originates from renal TECs, interstitial fibroblasts, vascular endothelial cells, and infiltrating immune cells in the kidneys. It is essential in multiple inflammatory diseases through its interaction with specific G protein-coupled receptors, namely, C-X-C motif chemokine receptor 1 (CXCR1) and CXCR2 [63]. The IL-8/CXCR1 signaling axis facilitates neutrophil recruitment and local inflammatory responses in kidney disease by enhancing the expression of intercellular adhesion molecule 1 (ICAM-1) and activating the p38 mitogen-activated protein kinase (MAPK) pathway [64]. The IL-8/CXCR2 axis induces mitochondrial dysfunction, which accelerates the senescence process and fibrosis in renal tubular cells, potentially underlying the pathogenesis of chronic kidney disease (CKD) [65]. Research indicates that plasma IL-8 levels in patients with sustained severe AKI may serve as effective biomarkers for predicting early postsurgical AKI (PS-AKI) in COVID-19 patients and are useful for forecasting the onset of AKI after cardiopulmonary bypass (CPB) surgery [66]. In a cisplatin-induced AKI mouse model, application of the CXCR1/2 antagonist G31P markedly reduced inflammatory cell infiltration in renal tissues, improved renal function, and mitigated tissue damage [67]. Moreover, in kidney transplant recipients, IL-8 levels in renal tissues markedly increase after ischemia–reperfusion injury (IRI), and the extent of elevation positively correlates with the duration of ischemia [68]. In kidney transplant recipients, the CXCR2-specific inhibitor repertaxin effectively prevented IRI-induced neutrophil infiltration and renal functional impairment. These animal model studies not only validated the phenomenon of elevated IL-8 expression in early AKI but also highlighted the importance of IL-8 as a prospective biomarker for forecasting death in mice [69,70]. Collectively, these findings indicate that IL-8 may function as an early warning biomarker for AKI and offer a theoretical and experimental framework for the formulation of novel therapeutic strategies targeting AKI (Fig. 2B and Fig. S4).

IL-12, a pivotal inflammatory mediator, is mostly released by macrophages, DCs, and B lymphocytes during immune activation [71]. It bridges innate and adaptive immunity by activating Th1 responses, thereby exacerbating the progression of inflammatory reactions. Song et al. [72] conducted research indicating that in kidney IRI, IL-12 critically regulates local inflammatory responses by facilitating DC maturation and enhancing the release of various key inflammatory mediators, including IL-6 and TNF-α. Further supporting this view, Padovani et al. [73] found substantially elevated levels of the proinflammatory cytokine IL-12 in a zebrafish larval model of cisplatin-induced AKI, indicating that IL-12 may be involved in the pathogenic mechanisms of chemically induced AKI. De Paiva et al. [74] demonstrated that IL-12 KO mice displayed resistance to IRI, indicating an important relationship between IL-12 and the development of ischemic renal injury. Linarin (LIN), a natural compound, has been validated through multiple in vitro and in vivo studies for its antioxidant and anti-inflammatory properties [75,76]. As demonstrated by Chengyu et al. [77] in in vivo experiments, LIN not only alleviates renal injury but also reduces the expression levels of the IL-12 p40 subunit, revealing that LIN may exert its renal protective effects by mitigating IL-12-mediated inflammatory responses (Fig. 2D and Fig. S6).

IL-17A (hereafter referred to as IL-17) transmits biological signals by binding to IL-17 receptor A (IL-17RA) and IL-17 receptor C (IL-17RC) [78]. A large-scale clinical study has shown that higher serum IL-17A levels are independently linked to a heightened risk of mortality and major adverse kidney events (MAKE) during the hospitalization of patients with AKI in the ICU [79]. In the pathogenesis of AKI, IL-17A functions through multiple distinct biological pathways [79–81]. Our prior research demonstrated that IL-17A elevates proinflammatory cytokine and chemokine levels in renal tissues, leading to neutrophil infiltration and apoptosis of TECs, which facilitates sepsis-associated AKI (SA-AKI). Renal function in SA-AKI mice with IL-17A KO was markedly improved, and the serum levels of TNF-α, IL-6, and IFN-γ were correspondingly reduced [82]. Naito et al. [80] similarly confirmed in a cecal ligation and puncture (CLP)-induced SA-AKI mouse model that exogenous IL-17A application exacerbated SA-AKI development, while IL-17A gene KO effectively decreased its incidence. In a cisplatin-induced AKI mouse model, the mRNA and protein expression levels of IL-17A were substantially elevated in the kidneys of wild-type (WT) mice [83]. In contrast, anti-IL-17A antibodies or IL-17A gene KO resulted in greater resistance to cisplatin-induced nephrotoxicity, confirming IL-17A’s critical role in cisplatin-induced AKI and indicating that antagonizing IL-17A offers substantial protective effects against this condition [83]. Studies on renal fibrosis following AKI in mouse models have revealed that IL-17A promotes the chemotactic aggregation of neutrophils and fibroblasts, thereby exacerbating renal fibrosis [84,85]. Conversely, inhibiting IL-17A using tamibarotene (commonly known as Am80) or neutralizing IL-17A with recombinant IL-17RC effectively mitigates post-AKI renal fibrosis [85,86]. Weng et al. [87] proposed that the TGF-β/Smad signaling pathway may be crucial in IL-17A-induced fibronectin expression, suggesting that intervention in this pathway may furnish a novel therapeutic approach to avoid kidney fibrosis. Given the multifaceted impact of IL-17A on AKI, future research should delve deeper into its specific mechanisms of action to better understand its importance in AKI and provide scientific grounds for developing new therapeutic strategies (Fig. 2E and Fig. S7).

IL-18, or interferon-γ inducing factor (IGIF) [88], is predominantly secreted by macrophages and is expressed in multiple cell types, such as renal TECs, intercalated cells, and tubulointerstitial cells. Recent studies have indicated that IL-18 not only serves as a common inflammatory marker but also plays a crucial role in the pathophysiology of AKI. Multiple studies indicate that IL-18 levels in the urine of patients with AKI are significantly elevated, making it a reliable marker for the onset of AKI within 12 h in critically ill patients [89]. IL-18 exacerbates AKI pathogenesis induced by sepsis, nephrotoxicity, and IRI [90]. Melnikov et al. [91] revealed that caspase-1-deficient mice, which lack the enzyme crucial for the mature forms of pro-IL-1β and pro-IL-18 cleavage, exhibit reduced neutrophil infiltration in the renal tissue following acute ischemia, thereby alleviating ischemic acute renal failure (ARF). A decrease in the kidneys’ conversion of pro-IL-18 to its mature form is correlated with the protective effect. Furthermore, by regulating inflammatory cell infiltration, the production of inflammatory cytokines and chemokines, and the conversion of bone marrow-derived M2-type macrophages into myofibroblasts, IL-18 plays a substantial role in the development of renal fibrosis after IRI [92]. In contrast, the inhibition of IL-18 can reduce the advancement of renal fibrosis following IRI [92], further underscoring the important role of IL-18 in AKI and its complications. Therefore, therapeutic strategies targeting IL-18 offer a promising approach for intervening in the pathological progression of AKI, providing a novel therapeutic target for its prevention and treatment (Fig. 2A and Fig. S1).

IL-24, or Mda-7, similar to IL-20, binds to 2 distinct receptor complexes: one consisting of IL-20R1 and IL-20R2, and the other formed by IL-22R1 and IL-20R2. Through these receptor pathways, IL-24 exerts broad biological functions including antitumor, antibacterial, and immunoregulatory properties [93]. The function of IL-24 in AKI has received recognition and attention in recent years [94–96]. Tabata et al. [94] observed in a mouse model of IRI-AKI that serum IL-24 levels significantly increased, preceding changes in serum creatinine levels. This indicates that IL-24 could function as an early biomarker for the onset of AKI. Additionally, with prolonged ischemic time, serum and urinary IL-24 levels showed an increasing trend, further supporting their potential as diagnostic indicators of AKI. Pap et al.’s [95] research demonstrated that IL-24 induces apoptosis in human kidney-2 (HK-2) cells and up-regulates the expression of TGF-β1, platelet-derived growth factor-B (PDGF-B), and connective tissue growth factor (CTGF). IL-24 is essential in facilitating apoptosis and fibrosis in renal TECs, likely via the activation of profibrotic signaling pathways. The kidneys of IRI-AKI animal models showed increased IL-24 expression, according to Schütte-Nütgen et al. [96], with renal TECs and infiltrating inflammatory cells serving as the main IL-24 sources. Their studies indicated that IL-24 not only induces apoptosis in renal TECs but also is associated with enhanced endoplasmic reticulum stress. Notably, IL-24 KO mice exhibited protective effects against renal damage and inflammatory responses, indicating a critical role for IL-24 in the pathogenesis of AKI [96]. Therefore, IL-24, as a potential biomarker, might be a promising therapeutic target and possible biomarker for people with AKI (Fig. 2C and Fig. S5).

IL-36, comprising 3 subtypes, IL-36α, IL-36β, and IL-36γ, is classified within the IL-1 superfamily of proinflammatory cytokines. These molecules interact with a heterodimer composed of the IL-36 receptor (IL-36R) and co-receptor acid phosphatase 5, tartrate resistant (ACP5), or transport inhibitor response 1 (TIR1), where proinflammatory chemokines and cytokines are produced through the activation of downstream signaling pathways. Traditionally, IL-36 is thought to primarily originate from cells of the adaptive immune system, such as T lymphocytes, B lymphocytes, and monocytes [97,98]. However, recent studies have revealed that renal tubular cells, interstitial cells, macrophages, and neutrophils infiltrating in tissues also express IL-36α [99], suggesting an underestimated role for IL-36 in innate immune responses. The up-regulation of IL-36α is associated with enhanced NF-κB activity and promotion of extracellular signal-regulated kinase (ERK) phosphorylation [100], 2 critical pathways involved in inflammation and the stress response. Clinically, patients with AKI have been shown to have higher amounts of IL-36α in their urine and more pronounced IL-36α staining has been detected in renal tissue biopsy samples. Animal model studies have further confirmed that following IRI, the mRNA and protein levels of IL-36α/β/γ are significantly increased in mouse renal tissues [99]. Experimental results showed that, compared to WT mice, IL-36R KO mice subjected to IRI had lower serum creatinine levels, lower blood urea nitrogen (BUN) levels, and lower mRNA levels of IL-6/TNF-α. Additionally, the protein expression of the NLRP3 inflammasome, IL-1β, and caspase 1 is suppressed [99]. This suggests that IL-36R participates in AKI pathogenesis by activating the NF-κB pathway and up-regulating IL-6 and TNF-α expression. Therefore, interventions targeting IL-36α and its receptor, IL-36R, may represent potential therapeutic targets for treating AKI, offering a new avenue for mitigating inflammatory responses (Fig. 2A and Fig. S1).

Additionally, cytokines such as IL-17C [10,101], IL-19 [102], IL-20 [103], IL-23 [104–106], and IL-34 [52,107] possess proinflammatory properties that can exacerbate the progression of AKI, although they have been less frequently reported in the current literature (Fig. 2C to G and Figs. S5 to S8).

## ILs Mitigating AKI Progression

**Fig. 3.**
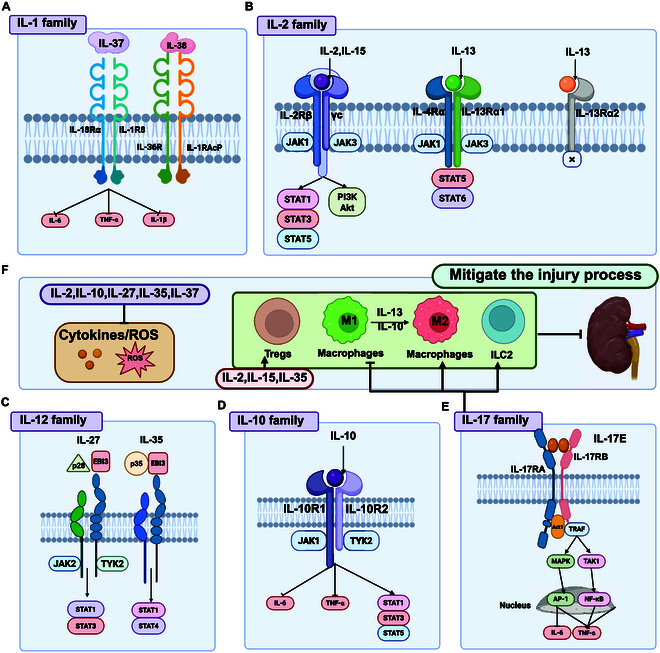
ILs mitigating AKI progression. This figure illustrates the mechanisms by which various IL families inhibit the progression of AKI. (A) IL-1 family: Signaling pathways involving IL-37, highlighting its interaction with IL-18Rα and IL-1R8 receptors. (B) IL-2 family: Signaling pathways of IL-2 and IL-15 through their interactions with IL-2Rβ and γc receptors, resulting in JAK1/JAK3 activation and downstream STAT1/STAT3/STAT5 and PI3K/Akt pathways. (C) IL-12 family: Signaling pathways of IL-27 and IL-35, showing their interactions with JAK2/TYK2 and STAT1/STAT3/STAT4 pathways. (D) IL-10 family: Role of IL-10 in inhibiting AKI progression through its interaction with IL-10R1 and IL-10R2 receptors, activating JAK1/TYK2 and downstream STAT1/STAT3/STAT5 pathways. (E) IL-17 family: Signaling pathways of IL-17E, showing its interaction with IL-17RA and IL-17RB receptors, leading to activation of TRAF, MAPK, TAK1, AP-1, and NF-κB pathways. (F) Overview of AKI inhibition: To summarize the overall process, cytokines such as IL-2, IL-10, IL-27, IL-35, and IL-37 mitigate inflammation by inhibiting pro-inflammatory cytokines and ROS. Meanwhile, IL-2, IL-15, and IL-35 regulate the development of Tregs, while IL-17E allows macrophages to change from a pro-inflammatory M1 phenotype to an anti-inflammatory M2 phenotype and ILC2s. These concerted actions collectively facilitate the resolution of inflammation and promote tissue repair. Figure created with BioRender.com.

IL-2, first cloned in 1983, not only encourages the growth of T cells but also is essential for controlling immunological responses [108]. The receptor comprises 3 distinct chains: Three IL-2 receptor chains are identified: IL-2 receptor α (IL-2Rα, CD25), IL-2 receptor β (IL-2Rβ, CD122), and IL-2 receptor γ (IL-2Rγ, CD132). The affinity of these receptors for IL-2 differs. Activated lymphocytes have IL-2Rα, which has a low affinity for IL-2. With a medium affinity for IL-2, IL-2Rβ and IL-2Rγ combine to form the IL-2Rβ/γ complex, which is mostly found on the surface of memory T cells and NK cells [109]. High-affinity binding of IL-2 occurs in the development of a 4-membered complex when activated T cells and regulatory Tregs coexpress the 3 receptors, IL-2R α/β/γ. The 3-dimensional (3D) structural study of this quaternary complex shows that IL-2 recruits IL-2Rβ and IL-2Rγ after initially forming an association with IL-2Rα. Medium- and high-affinity receptor types can convey IL-2 signals and carry out their specific tasks [110]. In IRI-AKI, inflammatory reactions typically play an important role [111,112]; mitigating inflammatory responses has become a critical component of various nonprotective strategies [111,113]. Tregs, a typical anti-inflammatory cell type, are essential for maintaining immune homeostasis [114,115]. In IRI, the depletion of Tregs exacerbates renal damage, whereas supplementation with Tregs protects the kidneys and mitigates injury [116,117]. Research indicates that IL-2 is essential for preserving the functionality of Tregs [118], and low-dose IL-2 has been proven to improve post-IRI renal conditions by enhancing endogenous Treg function [119]. Jang et al. [120] found that Tregs were significantly depleted in a cold IRI model following kidney transplantation in mice. Treatment with an IL-2/anti-IL-2 antibody complex (IL-2C) not only decreased reactive oxygen species (ROS)-mediated damage and improved renal antioxidant function but also decreased TEC apoptosis, facilitated renal regeneration, and alleviated the progression of chronic renal fibrosis subsequent to IRI, highlighting its potential for long-term renal protection and recovery. Furthermore, the fusion protein formed by combining IL-2 with IL-33, termed IL-233, exhibited enhanced renoprotective effects. The IL-233 fusion protein can mitigate inflammatory responses and prevent AKI induced by cisplatin, doxorubicin, and IRI by promoting Treg homeostasis and activation (Fig. 3B and Fig. S2) [121].

IL-10, secreted by Th2 cells in mice, can inhibit the production of IL-2 and IFN-γ [122,123]. Its biological effects are mediated through the classical signaling pathway, in which IL-10 binds to its receptor subunits IL-10Rα and IL-10Rβ on the surface of target cells, leading to the activation of intracellular Janus kinase 1 (JAK1) and tyrosine kinase 2 (TYK2). This activation subsequently triggers the phosphorylation of signal transducer and activator of transcription (STAT) proteins, including STAT3, STAT1, and STAT5, thereby mediating the anti-inflammatory and immunomodulatory effects of IL-10 [124]. As a key immunomodulatory factor, IL-10 can suppress the activity of neutrophils and monocytes and reduce the production of chemokines, cytokines, and nitric oxide, thereby attenuating inflammation and cytotoxic reactions linked to AKI [125]. Köken et al. [126] demonstrated that in an IRI mouse model, IL-10 treatment decreased the activation of oxidative stress indicators, such as superoxide dismutase (SOD) and catalase (CAT), in the kidney and prevented the decline in reduced glutathione (GSH) levels, indicating that IL-10 effectively prevents early oxidative damage caused by renal IRI. Deng et al. [127] discovered that IL-10 can prevent cisplatin- and ischemia-induced AKI by inflammatory cell activation and adhesion, as well as suppressing inducible nitric oxide synthase (iNOS) gene expression. In IL-10 (−/−) IRI mice, there was a notable increase in TNF-α, IL-6, and macrophage expression, resulting in more severe renal tissue injury and substantial impairment of renal function [128]. This further underscores the essential role of IL-10 in alleviating renal IRI damage. Recent studies indicate that the incorporation of IL-10 onto rectangular DNA origami nanostructures (rDONs) markedly improves the IL-10 accumulation and retention time in the kidneys. This modification facilitates the polarization of M1 macrophages into M2-type macrophages, leading to a notable decrease in proinflammatory factors and an increase in anti-inflammatory factors [129]. Recent studies have shown that loading IL-10 onto rDONs markedly enhances the accumulation and retention time of IL-10 in the kidneys; enabling it may efficiently polarize M1 macrophages into M2-type macrophages, which will raise anti-inflammatory molecules and drastically lower pro-inflammatory ones [129]. Milwid et al. [130] similarly demonstrated that mesenchymal stromal cells (MSCs), combined with pulsed focused ultrasound (pFUS) therapy, can up-regulate IFN-γ and stimulate the production of IL-10, thereby ameliorating cisplatin-induced AKI. Current evidence strongly supports the therapeutic potential of IL-10-targeted interventions in the clinical management of AKI (Fig. 3D and Fig. S5).

IL-15 is a 14- to 15-kDa secreted glycoprotein that signals through a complete heterotrimeric IL-15 receptor (IL-15R) composed of a high-affinity IL-15Ra subunit and an intermediate-affinity IL-15Rβγc heterodimer (IL-15Ra/IL-15Rβγc) [131]. Both IL-15 and its receptor are consistently expressed in normal renal TECs [132] and have been shown to enhance survival signaling in these cells through the JAK/STAT and PI3K/Akt pathways [133,134]. IL-15 can also activate MAPK and PI3K/Akt/mammalian target of rapamycin (mTOR) pathways. Eini et al. [135] elucidated that IL-15 activates anti-apoptotic signaling pathways in renal epithelial cells, an effect that was abrogated in IL-15Ra-deficient mice. Additionally, IL-15 mitigated cisplatin-induced apoptosis in renal TECs, and this protective effect was reversed in IL-15Ra-deficient renal epithelial cells. Finally, they observed that intrarenal IL-15 levels decreased 5.8-fold in SA-AKI, 11-fold in IRI-AKI, and 23-fold in cisplatin-induced AKI. In a cisplatin-induced AKI, IL-15 levels were inversely correlated with BUN levels. These studies indicate that IL-15 is a critical mediator for maintaining the normal function and survival of renal TECs (Fig. 3B and Fig. S2) [136].

IL-17E, or IL-25, exhibits the lowest amino acid sequence homology with IL-17A (~16%). IL-17E is generated by various cell types, such as T lymphocytes, eosinophils, mast cells, basophils, and intestinal and lung epithelial cells [137,138]. It has been associated to the etiology of tons of inflammatory illnesses, including asthma, chronic colitis, experimental autoimmune encephalomyelitis (EAE), and parasitic infections [138–140]. Recent studies have indicated that IL-17E exerts protective effects against kidney damage caused by IRI, nephrotoxic drugs, and obesity [11,141,142]. This protective mechanism likely mediates IL-17E-driven preservation of renal function and attenuation of histopathological injury in mice, potentially through enhanced activation of type 2 innate lymphoid cells (ILC2s) and multipotent progenitor type 2 cells (MPP2s). Concurrently, IL-17E promotes M2 macrophage polarization while suppressing M1 activation in renal tissues [11]. Therefore, the potential application of IL-17E in treating kidney diseases, particularly in mitigating kidney injury caused by IRI, drug toxicity, or obesity, warrants further clinical research and exploration (Fig. 3E and Fig. S7).

Moreover, cytokines including IL-13 [143], IL-27 [144], IL-35 [145], IL-37 [67], and IL-38 [146] are recognized as anti-inflammatory components of the IL family that can mitigate the progression of AKI, yet they have received relatively limited attention in the literature (Fig. 3B to F and Figs. S1, S2, and S6).

## ILs with Dual Functions

**Fig. 4.**
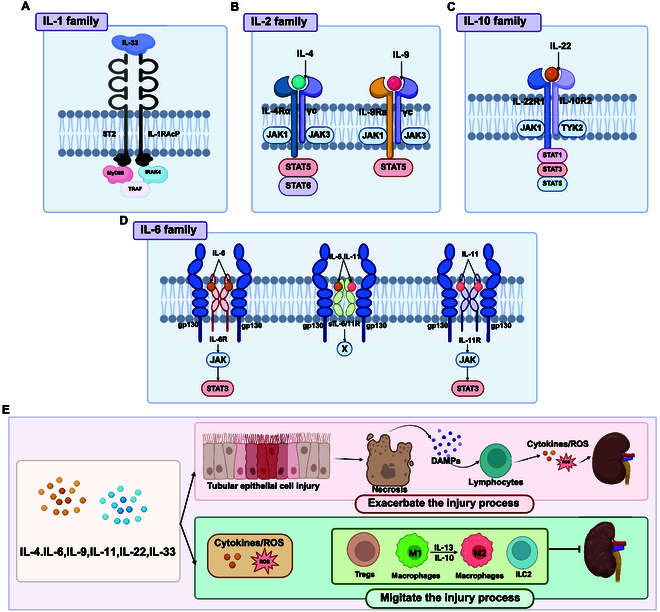
ILs with dual functions. This figure illustrates the mechanisms by which various IL families with dual functions influence AKI. (A) IL-1 family: Signaling pathways involving IL-33, highlighting its relationship to IL-1RacP and ST2 receptors, leading to activation of MYD88, IRAK4, and TRAF. (B) IL-2 family: Signaling pathways of IL-4 and IL-9 through their interactions with IL-4Rα and IL-9Rα receptors, respectively, leading to activation of JAK1/JAK3 and downstream STAT5/STAT6 pathways. (C) IL-10 family: Signaling pathway of IL-22 through its interaction with IL-22R1 and IL-10R2 receptors, leading to JAK1/TYK2 activation and downstream STAT1/STAT3/STAT5 pathways. (D) IL-6 family: Signaling pathways of IL-6, IL-11, and sIL-6R, showing their interactions with gp130 and IL-6R receptors, leading to activation of JAK/STAT3 pathways. (E) Overview of dual functions: The aforementioned cytokines such as IL-4, IL-6, IL-9, IL-11, IL-22, and IL-33 exert dual effects on the regulation of kidney injury. Figure created with BioRender.com.

IL-4, a pleiotropic type 2 cytokine, was initially discovered in 1982 as a 4-α helix bundle protein released by mast cells, CD4^+^ T cells, Th2 cells, basophils, and eosinophils [147]. IL-4 signals through either the IL-4Rα/IL-13Rα1 heterodimer or the IL-4Rα/γc heterodimer [148,149] and plays crucial roles in various immune and nonimmune functions [150]. Upon binding to its receptors, IL-4 activates multiple signaling pathways, such as insulin receptor substrate 2 (IRS2)/PI3K/Akt/mTOR and JAK-STAT. Notably, STAT6, a downstream component of these pathways, is enlisted and activated to mediate many of IL-4’s effects [151]. Research has established IL-4 as an important regulator in the recovery from renal tubular injury [141,152–154]. Zhang et al. [154] found that IL-4 promotes the M2 polarization macrophages in animal models of AKI, which is closely related to the repair of renal tubular damage. In IL-4/STAT6 KO mice, kidney damage is more severe in IRI-AKI models [155], underscoring IL-4’s protective role. Peruchetti et al. [156] confirmed that IL-4 offers protection against albumin overload-induced tubulointerstitial injury and that this effect is associated with the modulation of inflammatory responses. Furthermore, IL-4 can promote myeloid fibroblast activation by activating the JAK3/STAT6 signaling pathway [153,157], which is pivotal in renal fibrosis progression due to the recruitment of these cell precursors to the kidneys [158]. Liang et al. [152] established that IL-4Rα signaling is essential for the activation of bone marrow-derived fibroblast precursors in a murine model of folic acid (FA)-induced AKI renal fibrosis. Moreover, IL-4Rα deficiency results in inhibition of bone marrow-derived fibroblast activation and markedly reduces the progression of renal fibrosis, highlighting the potential therapeutic implications of targeting IL-4Rα in fibrotic kidney diseases. These studies imply that IL-4 plays 2 roles in the occurrence and progression of AKI (Fig. 4A and Fig. S2).

**Table 2.** Overview of the therapeutic potential of interleukin family members in AKI

Category	Name	Mechanism of action	Application in AKI	Reference
IL-1-related monoclonal antibodies	Anakinra	Recombinant human IL-1Ra binds IL-1 receptors, blocking IL-1 signaling	Alleviates sepsis-induced AKI, improves renal function by restoring GFR	[199]
	Rilonacept	Recombinant “IL-1 trap” fusion protein, binds and inactivates IL-1α/β, blocks signaling	Enhances cardiovascular health in high-risk CKD patients	[196]
	Canakinumab	Human anti-IL-1β antibody, blocks IL-1β receptor binding, inhibits inflammation, reduces serum IL-6	Reduces MACE in CKD patients, especially with strong anti-inflammatory response	[194,195]
IL-6-related monoclonal antibodies	Tocilizumab	Human anti-IL-6 antibody, blocks IL-6 signaling, reduces inflammatory cytokines, alleviates inflammation	Improves AKI pathology, reduces Scr and BUN, enhances patient survival	[201,208]
Binding proteins	RhIL-18BP	Binds IL-18, blocks IL-18R interaction, inhibits IL-18 activity	Mitigates renal inflammatory response, protects tubular and PTC cells, alleviates I/R injury	[92,198]
EVs	IL-10^+^ EVs	Delivers IL-10 to tubular cells via macrophage vesicles, inhibits mTOR, promotes mitochondrial health, drives M2 polarization	Engineered macrophages load IL-10 into EVs, treating ischemic AKI	[204]
	IL-37^+^ EVs	Delivers IL-37 via neutrophil vesicles, targets IRI endothelial cells, inhibits apoptosis, promotes proliferation	Neutrophil vesicles with IL-37 improve kidney IRI	[205]

IL-6 is a cytokine with several uses exhibiting both proinflammatory and anti-inflammatory properties [159]. In response to stimuli like TNF-α and IL-1β, renal resident cells—including TECs, podocytes, and mesangial cells—synthesize it [160–163]. From a mechanistic standpoint, IL-6 primarily mediates its effects through 2 signaling pathways: the classical membrane-bound IL-6 receptor (mbIL-6R/gp130) pathway and the trans-signaling pathway involving the soluble IL-6 receptor (sIL-6R/gp130). Both ultimately converge on the activation of the STAT3 signaling axis, which contributes to renal protective effects [164,165]. Andres-Hernando et al. [166] demonstrated that IL-6 participates in the formation of compensatory anti-inflammatory response syndrome (CARS) following AKI by enhancing IL-10 production in splenic macrophages, CD4^+^ T cells, and B cells, thereby effectively suppressing secondary inflammatory responses post-AKI. Studies have confirmed that IL-6 not only has the capacity to protect the kidney from further damage but also can promote harmful inflammatory reactions. This dual and context-dependent role has been experimentally validated across different AKI models. However, other studies have shown that in IRI-AKI mouse models, methyl-CpG binding protein 2 (MECP2) protects the kidneys from IRI by transcriptionally repressing IL-6/STAT3 signaling [167]. In models of AKI induced by IRI and HgCl2, the absence of IL-6 enhances renal function and reduces neutrophil infiltration [164,168]. These results suggest that IL-6 might have an important role in renal inflammation, deterioration of renal function, and glomerular structural damage, thus providing scientific evidence for understanding the dual role of IL-6 in renal tissue injury and protection (Fig. 4D and Fig. S3).

IL-22, belonging to the IL-10 family, exhibits substantial immunomodulatory processes and is classified as a pleiotropic cytokine [169]. It is primarily produced by innate lymphoid cells, Th17 cells, and Th22 cells [170]. In the kidney, its primary source is mononuclear phagocytes, which encompass various subpopulations of interstitial DCs and macrophages, along with proximal TECs (PTECs). The receptor for IL-22 is specifically expressed in renal TECs [13,171,172]. Research indicates that IL-22 promotes the repair and anti-apoptotic properties of renal TECs, thereby protecting the kidney function. Kulkarni et al. [172] found that in AKI mouse models, necrotic tubular cells and oxidative stress selectively induced IL-22 secretion via the Toll-like receptor 4 (TLR4) pathway, activating its receptor and accelerating TEC regeneration and repair through the JAK/STAT3 and ERK1/2 signaling pathways. Additionally, high concentrations of IL-22 have been shown to prevent ischemic AKI, diabetic nephropathy, and acetaminophen-induced renal injury [56,171]. It is yet unclear how IL-22 contributes to acute renal damage and kidney disease. Other studies indicate that elevated IL-22 levels alone do not directly cause renal injury but can further activate the DNA damage response (DDR) and facilitate apoptosis in simulations of renal injury induced by cisplatin or AA [13]. When downstream STAT3 or p53 is inhibited, an anti-apoptotic effect similar to that observed in IL-22 and IL-22RA1 KO models can be mimicked, and complete KO of IL-22 markedly prevents cisplatin- or AA-induced AKI [13]. (Fig. 4C and Fig. S5).

IL-33, referred to as IL-1F11/nuclear factor from high endothelial venules (NF-HEV), has a notable proinflammatory function and is localized in the nuclei of endothelial cells, epithelial cells, and fibroblasts in diverse tissues [173–175]. It acts as an alarmin that is rapidly released following cell necrosis to activate nonimmune and innate immune cells, thereby promoting the secretion of proinflammatory cytokines [175,176]. Additionally, IL-33 activates inflammatory pathways that are dependent on myeloid differentiation primary response gene 88 (MyD88) by binding to its specific receptor, IL-1R-like 1 (ST2), and its co-receptor, IL-1RAcP [177,178]. Lately, the function of IL-33 in mediating kidney diseases has gained recognition [179], with its exacerbating effects on AKI confirmed in multiple mouse models [173,180,181]. IL-33 inhibition improved renal injury and fibrosis induced by IRI, as it reduced recruitment of bone marrow-derived fibroblasts, decreased inflammatory cell infiltration, and lowered proinflammatory cytokines and chemokines [181]. In cisplatin-induced AKI mouse models, neutralizing IL-33 therapy can reduce CD4^+^ T cell infiltration in the kidneys, lowered serum creatinine levels, and alleviate acute tubular necrosis (ATN) and cellular apoptosis. Park et al. [182] further demonstrated that anti-IL-33 therapy ameliorated ovalbumin (OVA)-induced AKI in a murine model. In contrast, administration of recombinant IL-33 exacerbated these pathological features, indicating that IL-33 modulation plays an important role in the progression of AKI. These studies suggest that blocking the IL-33 signaling pathway may offer a novel therapeutic strategy for AKI [173]. However, several studies have indicated that IL-33 protects against AKI [14,183]. IL-33 functions as a nuclear component by chelating nuclear NF-κB, which diminishes NF-κB-mediated gene expression and subsequently mitigates inflammatory responses [184]. Cao et al. [14] showed that IL-33 promotes the growth of ILC2, M2 macrophages, and Tregs and initiates Th2 immune responses, thereby providing renal protection. Moreover, this study suggests that the beneficial effects of IL-33 observed in humanized mouse models of renal IRI may be applicable to humans [14]. The diverse biological effects of IL-33 may be related to its dose, timing of action, and form (e.g., nuclear factor or cytokine) [14,185] (Fig. 4A and Fig. S1).

Furthermore, ILs, such as IL-9 [186–188] and IL-11 [189,190], also exhibit dual regulatory effects on AKI progression; however, they have received comparatively little attention in the literature (Fig. 4A and Figs. S2 and S3).

## ILs in the Pathophysiological Progression from AKI to CKD

During the pathological transition from AKI to CKD, the IL family undergoes a functional evolution from mediating acute injury responses to promoting chronic fibrotic remodeling. IL-1β has been shown to induce a senescent phenotype in renal PTECs under hypoxic conditions, characterized by reduced proliferation, G2/M cell cycle arrest, up-regulated expression of p21, increased activity of senescence-associated β-galactosidase (SA-β-gal), and enhanced production of pro-inflammatory and pro-fibrotic SASP factors [54]. IL-18 plays a pivotal role in the development of renal fibrosis following IRI, primarily by modulating inflammatory cell infiltration as well as cytokine and chemokine production and by promoting the transformation of bone marrow-derived M2 macrophages into myofibroblasts. Inhibition of IL-18 has been demonstrated to attenuate the progression of renal fibrosis post-IRI [92]. In a FA-induced AKI mouse model, genetic deletion of IL-18 markedly reduces the expression levels of key regulators of epithelial–mesenchymal transition (EMT), including TGF-β1 and vimentin, along with a marked decrease in collagen type I (COL-1), a hallmark marker of renal fibrosis, further highlighting the profibrotic role of IL-18 in the AKI-to-CKD transition [191]. Notably, IL-6, which possesses dual functional properties, appears to predominantly promote fibrosis during this stage. Studies have shown that specific blockade of IL-6 trans-signaling in a murine model of IRI-induced AKI to CKD not only accelerates renal tissue repair but also significantly reduces ECM deposition and α-smooth muscle actin (α-SMA) expression, suggesting a protective effect of IL-6 inhibition against the progression of chronic renal fibrosis induced by IRI [12]. The IL-33 signaling pathway also contributes to fibrosis during the AKI-to-CKD transition by regulating the accumulation of myeloid fibroblast precursors, inflammatory cell infiltration, and the expression of pro-inflammatory cytokines and chemokines [181]. Intriguingly, pretreatment with IL-33 before unilateral ureteral obstruction (UUO) has been reported to expand renal ILC2s and Tregs and to ameliorate renal fibrosis [192]. However, such antifibrotic effects of IL-33 have not yet been observed in models of AKI to CKD. In contrast, several protective cytokines maintain a dynamic balance to counteract fibrotic processes. IL-10 exerts anti-inflammatory and anti-fibrotic effects by suppressing CD4^+^ T cell proliferation and M1 macrophage polarization [50]. IL-22, on the other hand, protects against TGF-β1-induced renal fibrosis and inflammation by activating the Jagged1/Notch1 signaling pathway, thereby effectively delaying the pathological progression of AKI to CKD in vitro [193]. Collectively, these findings highlight the critical roles of ILs in driving the AKI-to-CKD transition through dynamic functional shifts, underscoring their potential as therapeutic targets for interrupting or modifying the long-term consequences of AKI.

## Therapeutic Potential of IL Family and Related Monoclonal Antibodies in AKI

### IL-targeted therapies for AKI

Targeted therapy based on the IL family signaling pathways has become a new direction for the intervention of AKI. Within the IL-1 family, canakinumab, a humanized monoclonal antibody that specifically neutralizes IL-1β, provides a mechanistic foundation for its application in AKI based on research accumulated in CKD studies [194,195]. Rilonacept, a fusion protein capable of neutralizing both IL-1α and IL-1β, has been demonstrated to have therapeutic value in AKI [196]. Notably, targeted therapy against the IL-17 family has shown outstanding efficacy in suppressing renal inflammation; IL-17A neutralizing antibodies can substantially reduce the infiltration of polymorphonuclear neutrophils and macrophages in ischemic AKI models [197], while IL-17C antibodies not only inhibit Th17 cell activation but also reduce the expression of pro-inflammatory cytokines [10]. Regarding the mechanisms of apoptosis and fibrosis, the natural IL-18 inhibitor IL-18BP has demonstrated dual protective effects in ischemia models, as it can decrease renal tubular cell apoptosis and delay the progression of renal fibrosis [92,198]. In addition, modulation of the IL-22 signaling pathway has been found to specifically alleviate cisplatin-induced nephrotoxic injury [13]. In the field of complication prevention, IL-33 monoclonal antibodies effectively prevent secondary cardiac damage following AKI by blocking myocardial lesion-related pathways [57]. These targeted therapeutic strategies not only curb AKI progression by blocking key inflammatory mediators but also provide novel insights into improving organ crosstalk and long-term prognosis.

### IL receptor-targeted therapies for AKI

Targeted intervention strategies against IL receptors demonstrate several advantages compared to directly targeting the cytokines themselves, including broad-spectrum blockade of multiple pathogenic ligands (such as IL-1α/β), comprehensive termination of downstream signaling pathways, and more convenient clinical administration. IL-1R antagonist (IL-1Ra) and anakinra (recombinant human IL-1R antagonist) competitively occupy the IL-1R binding site, thereby blocking the binding of IL-1α and IL-1β to the receptor and inhibiting their associated intracellular signaling pathways [199,200]. Although studies suggest that the combination of anakinra with zinc may increase the risk of AKI, it has also shown important therapeutic effects in alleviating sepsis-induced AKI and improving renal function [199]. The humanized monoclonal antibody targeting the IL-6 receptor tocilizumab (TCZ) not only exerts protective effects in sepsis-induced AKI and improves patient survival rates [201] but also delays cellular senescence in rhabdomyolysis-associated AKI by inhibiting the IL-6/GATA2/SERPINE1 pathway and down-regulates the mRNA and protein levels of cell cycle regulatory proteins P53 and P21 [202]. In addition, basiliximab, an IL-2 receptor-targeted agent, when combined with a delayed tacrolimus administration protocol, substantially reduces the incidence of AKI in the early period following liver transplantation [203]. These studies provide new directions for precise intervention in AKI, and future research may further explore receptor-targeted therapeutic strategies to more comprehensively improve clinical outcomes in patients with AKI.

### Extracellular vesicles in AKI therapy

IL-10 is an important cytokine that inhibits inflammation and controls responses by preventing the production of proinflammatory factors and promoting anti-inflammatory mechanisms. Recent studies indicate that engineered macrophages producing extracellular vesicles (EVs) containing IL-10 can effectively address ischemic AKI [204]. This therapy enhances the stability and targeting of IL-10 while reducing the risk of progression to CKD. Moreover, IL-10 has the capacity to inhibit the onset and progression of AKI by down-regulating systemic or local inflammatory responses, indicating its potential as an AKI treatment target.

In order to avoid the potential adverse consequences of neutrophil-derived natural EVs and to administer IL-37 in a promising manner, Goto et al. [205] developed a new engineering technique for the quick and easy creation of neutrophil membrane-derived vesicles (N-MVs). N-MVs improved IL-37 stability and its targeted distribution to injured IRI kidney endothelial cells through P-selectin glycoprotein ligand-1 (PSGL-1). N-MVs encapsulated in IL-37 have been shown to improve renal IRI in vitro and in vivo by promoting endothelial multiplication of cells and angiogenesis, inhibiting endothelial cell death, and lowering leukocyte infiltration and the synthesis of inflammatory factors (Table 2).

## Summary and Outlook

Over the past 3 decades, research on ILs has shifted from a singular cytokine paradigm to an appreciation of regulatory networks. This review comprehensively elucidates the diversity and complexity of roles that ILs play in the pathogenesis of AKI. On the one hand, ILs form a balanced network of pro-inflammatory and anti-inflammatory responses, as well as injury and repair processes, by orchestrating the activation of immune cells and the crosstalk between these cells and renal parenchymal cells. On the other hand, their functionality exhibits marked cell-specific characteristics. For instance, the activation of IL-1R1 in renal TECs accelerates epithelial dysfunction and apoptosis via the NF-κB pathway. Conversely, IL-1R1 signaling in endothelial cells enhances toxin-induced AKI recovery by restoring VEGFA-dependent endothelial cell viability and density. In podocytes, IL-1R1 activation stimulates intracellular Akt signaling cascades, thereby ameliorating NTS- and doxorubicin-induced podocyte injuries.

The application prospects of ILs in AKI therapy are particularly promising. Targeted therapies focusing on specific ILs or their receptors, alongside advancements in EV technology, may offer novel therapeutic options for AKI. A critical factor in realizing these therapeutic strategies lies in gaining a deeper understanding of the precise mechanisms through which ILs operate within AKI, and how they mediate crosstalk among renal resident cells, immune cells, and fibroblasts. Furthermore, given the comprehensive management requirements for AKI patients, future investigations should actively explore the role of ILs in the progression from AKI to CKD and in remote organ complications. Through these endeavors, we can aspire to develop innovative treatment approaches for AKI, providing solutions to global health challenges and bringing new hope to AKI patients.
