# Supplementary material for: Mechanisms Underlying the Impact of Interleukin Family on Acute Kidney Injury: Pathogenesis, Progression, and Therapy
Source: Research (Wash D C). 2025 Jun 13;8:0738. doi: 10.34133/research.0738 (PMC12163381; doi:10.34133/research.0738)
Supplement: Supplementary 1 — Figs. S1 to S8 [file research.0738.f1.docx]

**
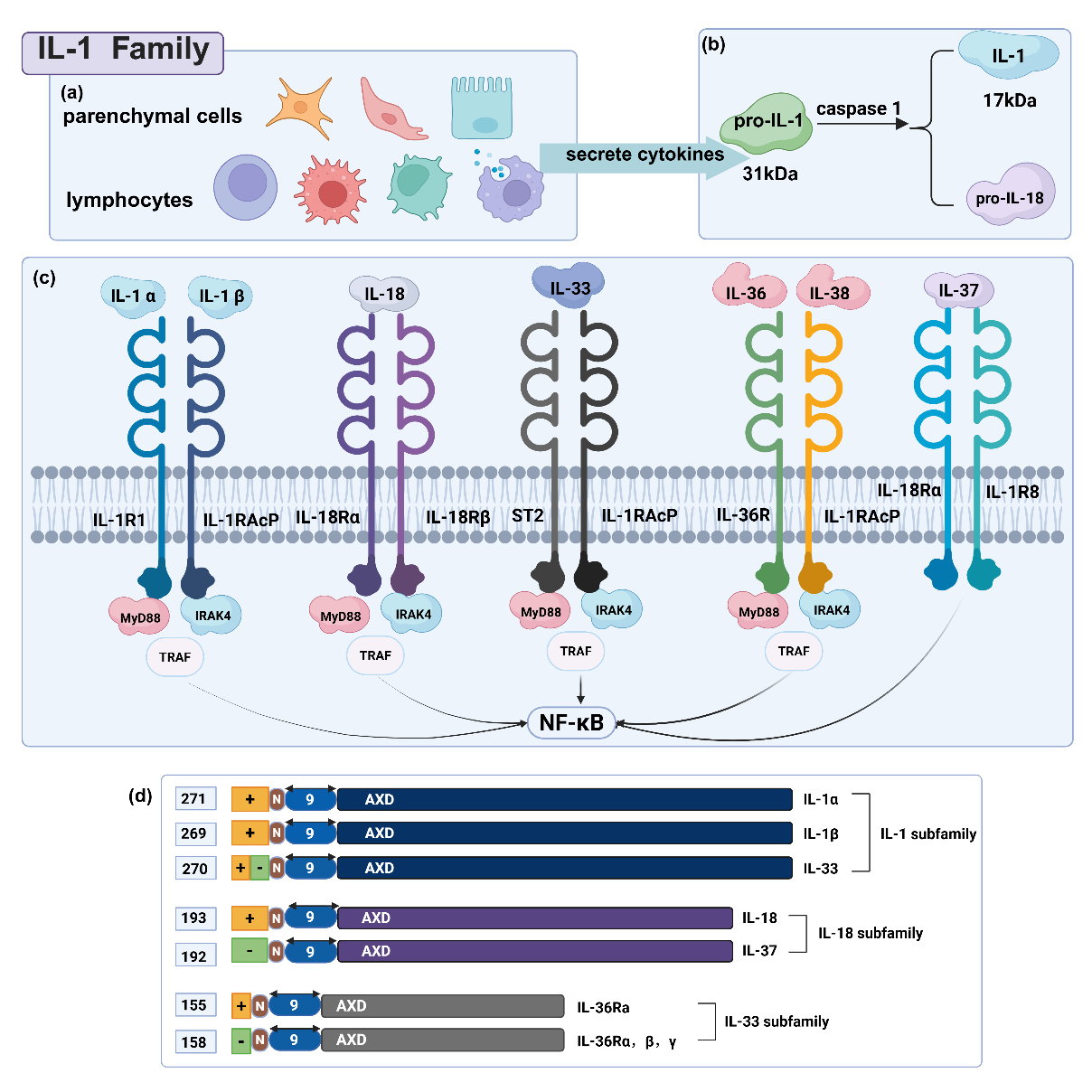
SUPPLEMENTARY MATERIALS**

***Figure S1*. IL-1 Family: (a) Sources of Cytokines in the IL-1 Family.** This illustrating the secretion of cytokines by parenchymal cells and lymphocytes. An organ's parenchymal cells are its functioning cells (such as hepatocytes in the liver or cardiomyocytes in the heart), and lymphocytes, which are key elements that make up the immune system, both help bring about the release of cytokines. **(b) Processing of Pro-IL-1 and Pro-IL-18 by Caspase 1.** This panel shows the processing of pro-IL-1 and pro-IL-18 by caspase 1. Pro-IL-1 is broken down by caspase 1 into its active form (17 kDa), while pro-IL-18 is similarly processed. The active forms of these cytokines are essential for inflammation and immunological reaction. **(c) Signaling Pathways of the IL-1 Family Cytokines.** This diagram details the signaling pathways of various interleukins within the IL-1 family. It includes **IL-1α and IL-1β**, which interact with IL-1R1 and IL-1RAcP, resulting in the starting up of MyD88, IRAK4, TRAF, and ultimately NF-κB; **IL-18**, which interacts with IL-18Rα and IL-18Rβ, activating comparable downstream signaling molecules;**IL-33**, which connects to ST2 and IL-1RAcP, activating the same signaling pathway; **IL-36**, which interacts with IL-1Rrp2 and IL-1RAcP; **and IL-37**, which interacts with IL-18Rα and IL-1R8. **(d) Structural Features and Subfamilies of IL-1 Family Members.** This section offers a thorough breakdown of the structural features and subfamilies of IL-1 family members: **IL-1α, IL-1β, and IL-33** belong to the IL-1 subfamily; **IL-18 and IL-37** belong to the IL-18 subfamily; **IL-36Ra, IL-36β, and IL-36γ** belong to the IL-33 subfamily. Each member is characterized by specific domains and motifs that determine their functions and interactions with receptors. Figure created with BioRender.com.


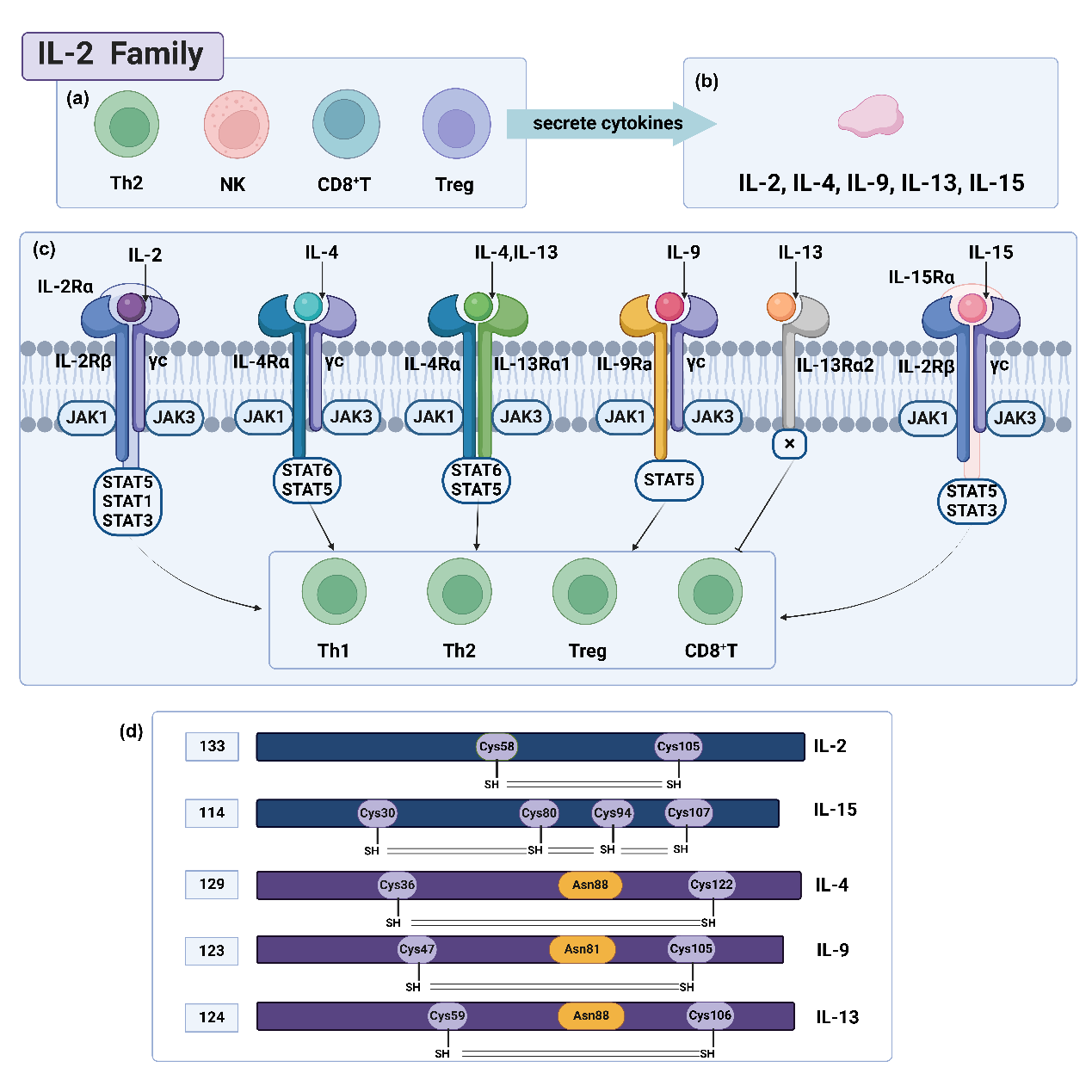
***Figure S2*. IL-2 Family: (a)** **Sources of Cytokines in the IL-2 Family.** This section delineates the major functional classes of leukocytes that participate in mediating immunological reactions. These cells include Th2, NK, CD8^+^T, and Treg subtypes. They secrete cytokines—signaling molecules essential for immune cell communication and functioning. **(b) IL-2 Family Cytokines Secreted.** This section delineates cytokine profiles of the immune cell subtypes characterized in Section A. The cytokines listed are IL-2, IL-4, IL-9, IL-13, and IL-15. These cytokines support cell proliferation, differentiation, and activation, among other immune system processes. **(c) IL-2 Family** **Cytokine receptor signaling pathways.** This diagram shows the interactions between different cytokines and their corresponding receptors on the outside of the immune cells. This includes the binding of IL-2, IL-4, IL-9, IL-13, and IL-15 to their receptors, which leads to the turning on of intracellular routes for signaling involving JAK/STAT proteins. The downstream effects of these pathways result in the distinction and activation of Th1, Th2, Treg, and CD8^+^ T-cells. **(d)** **Structural Diagrams of IL-2 Family.** This section provides structural diagrams of the cytokines IL-2, IL-15, IL-4, IL-9, and IL-13. Each diagram highlights specific amino acid positions and disulfide bonds that are critical for these proteins' composition and roles. In the tertiary structure, IL-2's β-sheet domain contains Cys58 and Cys105, whereas IL-15's α-helical region harbors four conserved cysteines (C30, C80, C94, C107). These structural features are important for the biological activities of cytokines. This detailed description should help understand the complex interactions and roles of cytokines in the immune system, as depicted in the figure. Figure created with BioRender.com.


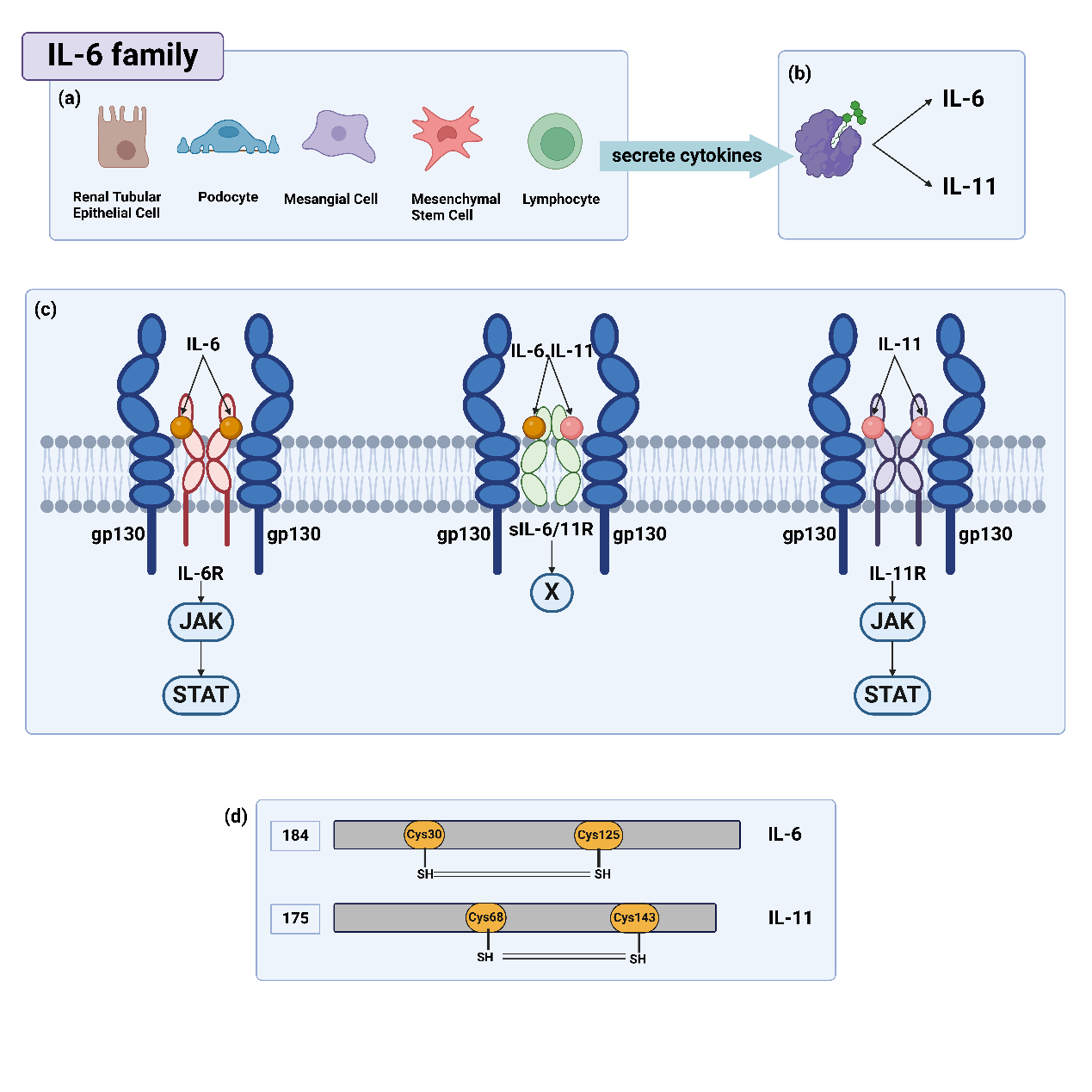
***Figure S3*. IL-6 Family: (a)** **Sources of Cytokines in the IL-6 Family.** This section illustrates various cell types that secrete cytokines, including podocytes, renal tubular epithelial cells, mesangial cells, mesenchymal stem cells, and lymphocytes. **(b) IL-6 Family Cytokines** **Secreted.** This section highlights the specific cytokines secreted by immune cells, specifically IL-6 and IL-11. **(c) IL-6 Family Cytokine Receptor Signaling Pathways.** This diagram shows the interactions between different cytokines and their respective receptors on the surface of immune cells. This includes the binding of IL-6 and IL-11 to their receptors, which leads to the activation of intracellular signaling pathways involving JAK/STAT proteins. **(d)** **Structural Diagrams of IL-6 Family.** This section provides structural diagrams of the cytokines IL-6 and IL-11. Each schematic emphasizes key residues and disulfide bonds pivotal to protein structural integrity and functional activity. For instance, IL-6 features Cys30 and Cys125 residues, whereas IL-11 contains analogous disulfide-forming residues at positions 68 and 143. This in-depth examination clarifies the multifaceted signaling networks and functional contributions of cytokines within immune regulation, as illustrated in the accompanying diagram. Figure created with BioRender.com.

**
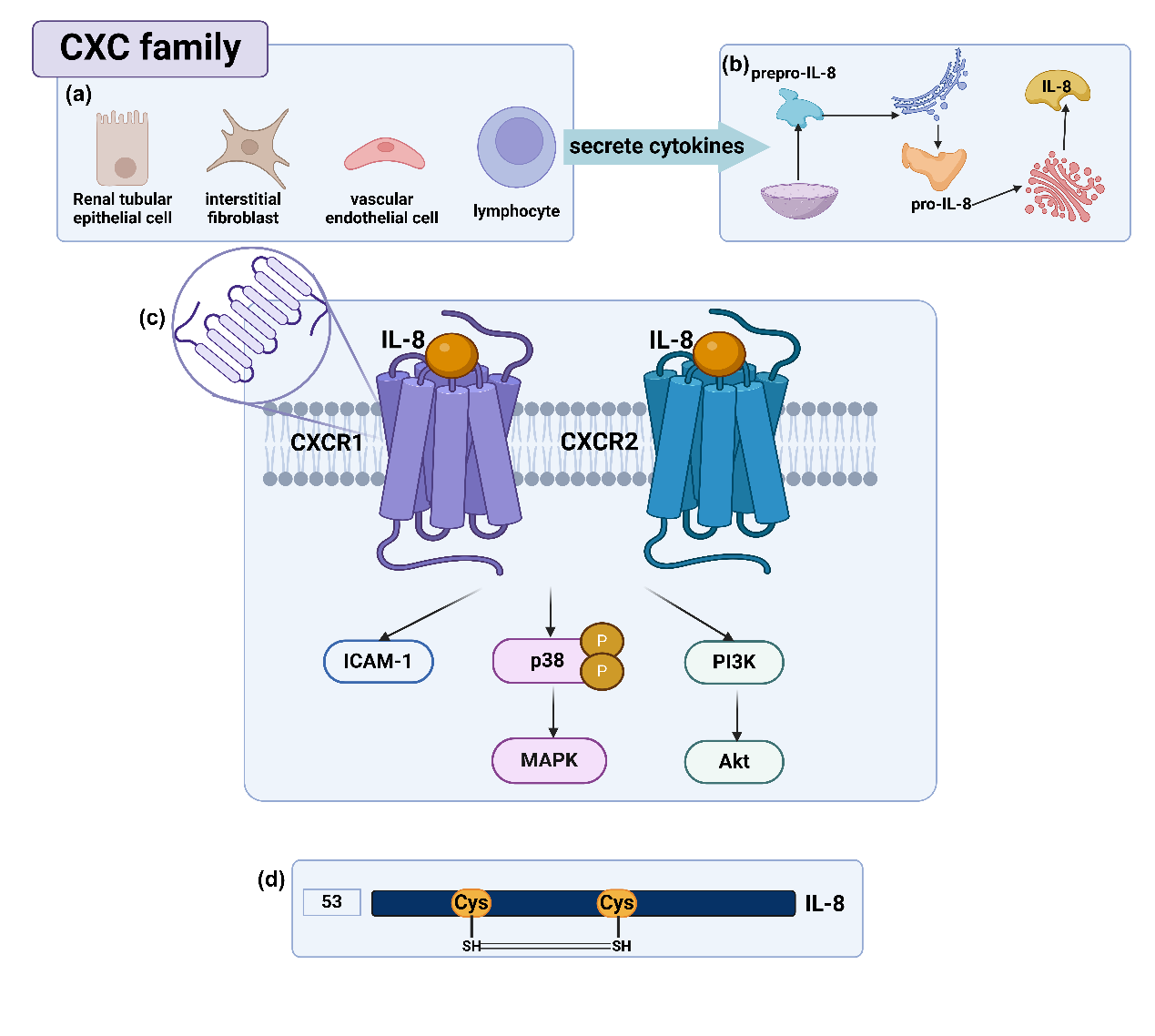
*Figure S4*. CXC Family: (a) Sources of Cytokines in the CXC Family.** This section illustrates various cell types that secrete cytokines, including Renal Tubular Epithelial Cells, Interstitial Fibroblasts, Vascular Endothelial Cells, and Lymphocytes. **(b) IL-8 Processing and Secretion.** This section highlights the processing and secretion of IL-8. It shows the conversion from prepro-IL-8 to pro-IL-8 and finally to mature IL-8. **(c) CXC Family Cytokine Receptor Signaling Pathways.** This diagram shows the interaction between IL-8 and its receptors CXCR1 and CXCR2 on the surface of the immune cells. The binding of IL-8 to these receptors leads to the activation of intracellular signaling pathways involving ICAM-1, p38 MAPK, PI3K, and Akt. **(d)** **Structural Diagram of IL-8.** This section provides a structural diagram of the cytokine IL-8, highlighting specific amino acid positions and disulfide bonds that are critical for its structure and function. The diagram highlights Cys53 and Cys72 residues linked by a disulfide bond. This comprehensive analysis elucidates the intricate regulatory mechanisms of cytokines within the immune system, as outlined in the accompanying schematic. Figure created with BioRender.com.


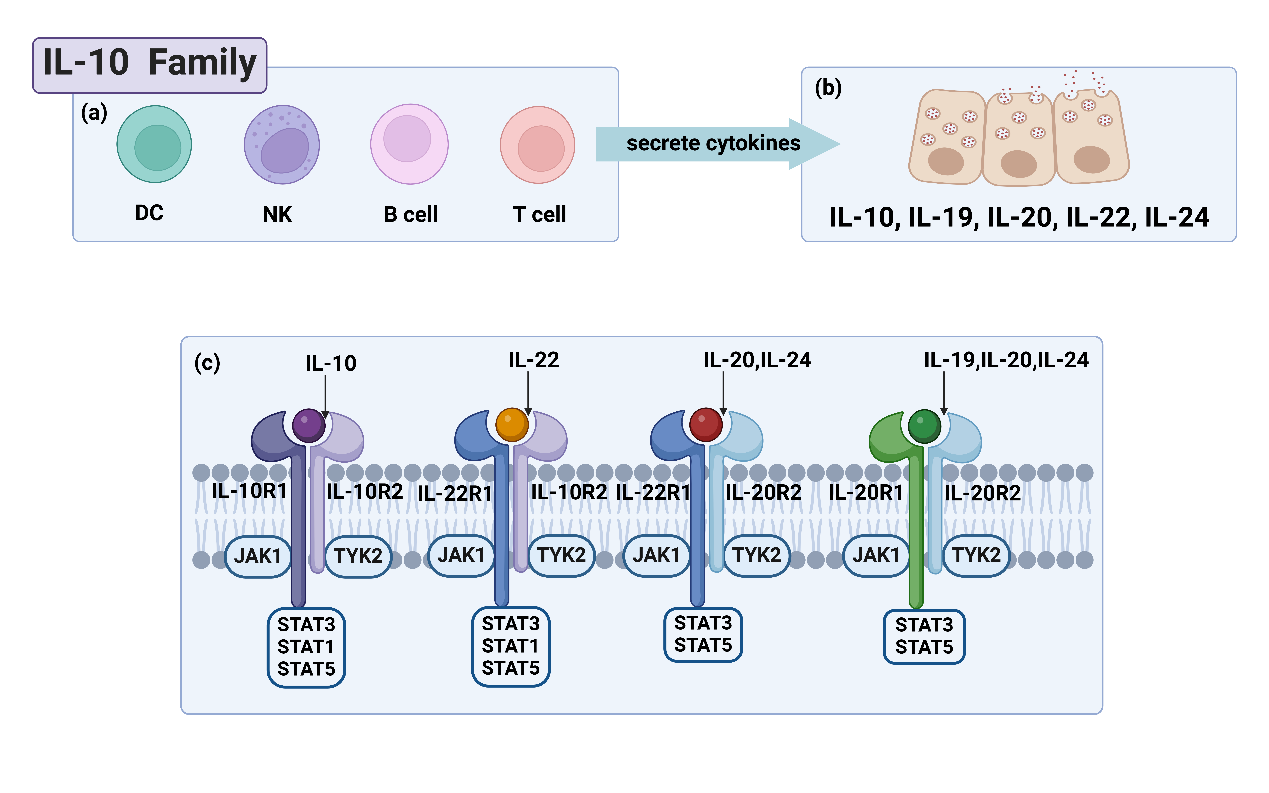
***Figure S5*. IL-10 Family: (a)** **Sources of Cytokines in the IL-10 Family.** This section illustrates various immune cells that secrete cytokines, including NK cells, B cells, T cells, and DC. **(b) IL-10 Family Cytokines** **Secreted.** This section highlights the specific cytokines secreted by the immune cells mentioned in Section A. The cytokines listed are IL-10, IL-19, IL-20, IL-22, and IL-24. **(c) IL-10 Family Cytokine Receptor Signaling Pathways.** This diagram shows the interactions between different cytokines and their respective immune cell surface receptors. This involves cytokine-receptor binding, initiating intracellular signaling cascades mediated by JAK/STAT proteins. Specifically, IL-10 binds to IL-10R1 and IL-10R2, activating JAK1 and TYK2, which subsequently phosphorylate STAT3, STAT1, and STAT5.IL-22 binds to IL-22R1 and IL-10R2, activating JAK1 and TYK2, which in turn activate STAT3, STAT1, and STAT5.IL-20 and IL-24 bind to IL-20R1 and IL-20R2, activating JAK1 and TYK2, which in turn activate STAT3 and STAT5, respectively.IL-19, IL-20, and IL-24 bind to IL-20R1 and IL-20R2, activating JAK1 and TYK2, which in turn activate STAT3 and STAT, respectively5. This comprehensive analysis clarifies the intricate regulatory roles and interactions of cytokines in immune system dynamics, as outlined in the accompanying schematic. Figure created with BioRender.com.

**
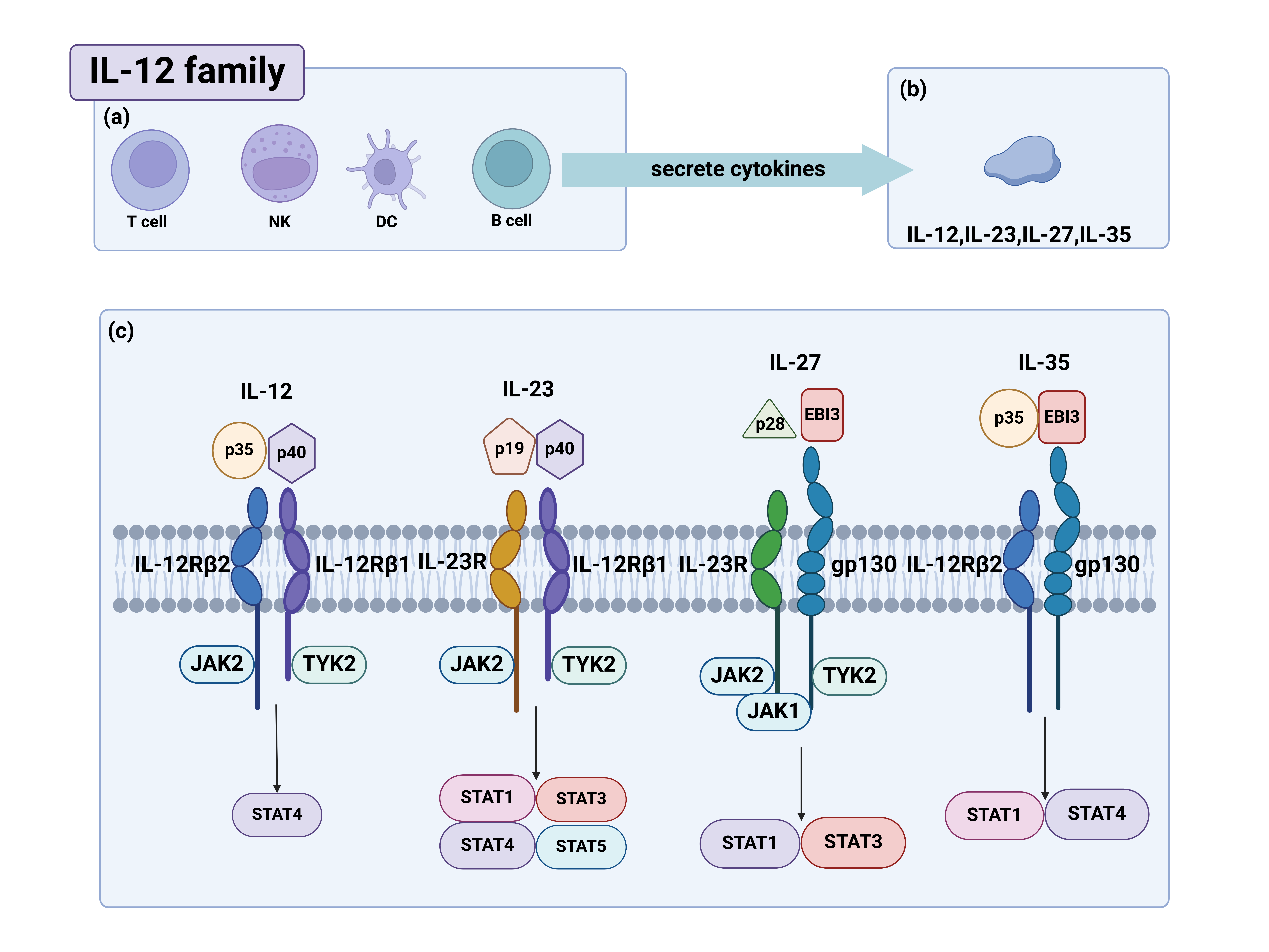
*Figure S6*. IL-12 Family: (a) IL-12 Family Cytokines** **Secreted.** This section illustrates various immune cells that secrete cytokines, including NK cells, DC, T cells, and B cells. **(b) IL-12 Family Cytokines** **Secreted.** This section highlights the specific cytokines secreted by the immune cells mentioned in Section A. The listed cytokines were IL-12, IL-23, IL-27, and IL-35. **(c) IL-12 Family Cytokine Receptor Signaling Pathways.** This schematic delineates interactions among specific cytokines and their corresponding immune cell surface receptors. This involves cytokine-receptor binding, initiating intracellular signaling cascades mediated by JAK/STAT proteins. Specifically, IL-12: Binds to the heterodimeric receptor complex IL-12Rβ1/IL-12Rβ2, initiating JAK2 and TYK2 kinase activity. This cascade phosphorylates and activates STAT4.IL-23 Engages IL-12Rβ1 paired with IL-23R, stimulating JAK2/TYK2-mediated signaling. Activated STAT1, STAT3, STAT4, and STAT5. IL-27 signals through the gp130/IL-12Rβ2 receptor complex, recruiting JAK1, JAK2, and TYK2. Phosphorylated STAT1 and STAT3 modulate immune tolerance and antiviral responses.IL-35 utilizes the gp130/IL-12Rβ2 receptor pair to activate JAK1/JAK2/TYK2 kinases, leading to STAT1 and STAT4 activation. This comprehensive analysis elucidates the intricate regulatory mechanisms of cytokines within the immune system, as demonstrated in the accompanying schematic. Figure created with BioRender.com.

**
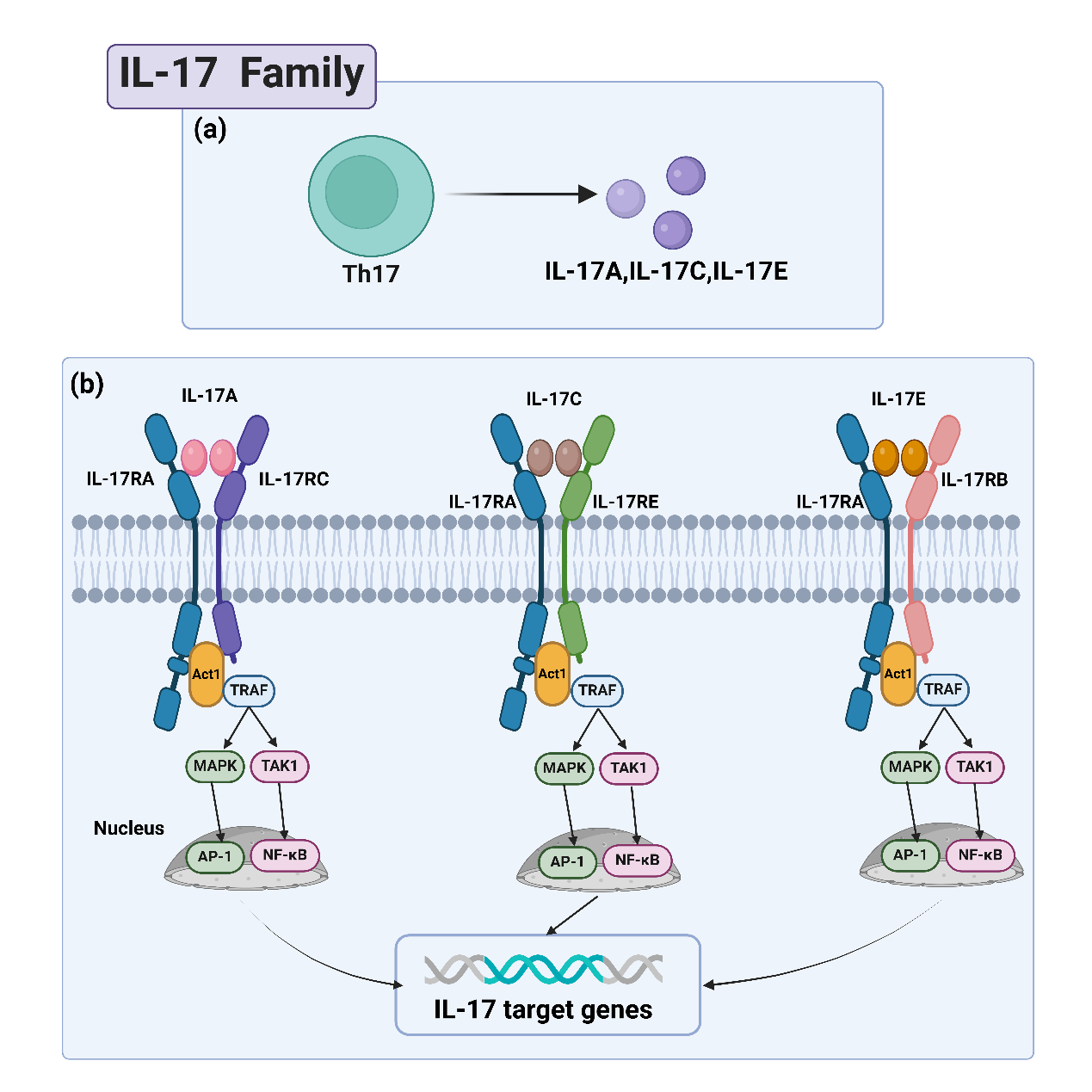
*Figure S7*. IL-17 Family: (a) IL-17 Family Cytokines** **Secreted.** Th17 cells secrete IL-17 family cytokines, such as IL-17A, IL-17C, and IL-17E. **(b) IL-12 Family Cytokines** **Secreted. IL-17A:** Binds to IL-17RA and IL-17RC, activating Act1 and TRAF, which in turn activate MAPK and TAK1. These pathways trigger nuclear translocation and activation of AP-1 and NF-κB, resulting in the transcription of IL-17 target genes. **IL-17C:** Binds to IL-17RA and IL-17RE, activating Act1 and TRAF, which in turn activate MAPK and TAK1. These pathways lead to the activation of AP-1 and NF-κB in the nucleus, resulting in the transcription of IL-17 target genes. **IL-17E:** IL-17 binds to its heterodimeric receptors, IL-17RA and IL-17RE, initiating Act1 and TRAF6 recruitment. These adaptor proteins activate the MAPK and TAK1 signaling cascades, which ultimately drive the nuclear translocation of AP-1 and NF-κB, inducing the transcription of IL-17-responsive genes. This comprehensive analysis elucidates the regulatory dynamics of cytokines within the immune system, as illustrated in the accompanying schematic. Figure created with BioRender.com.

***
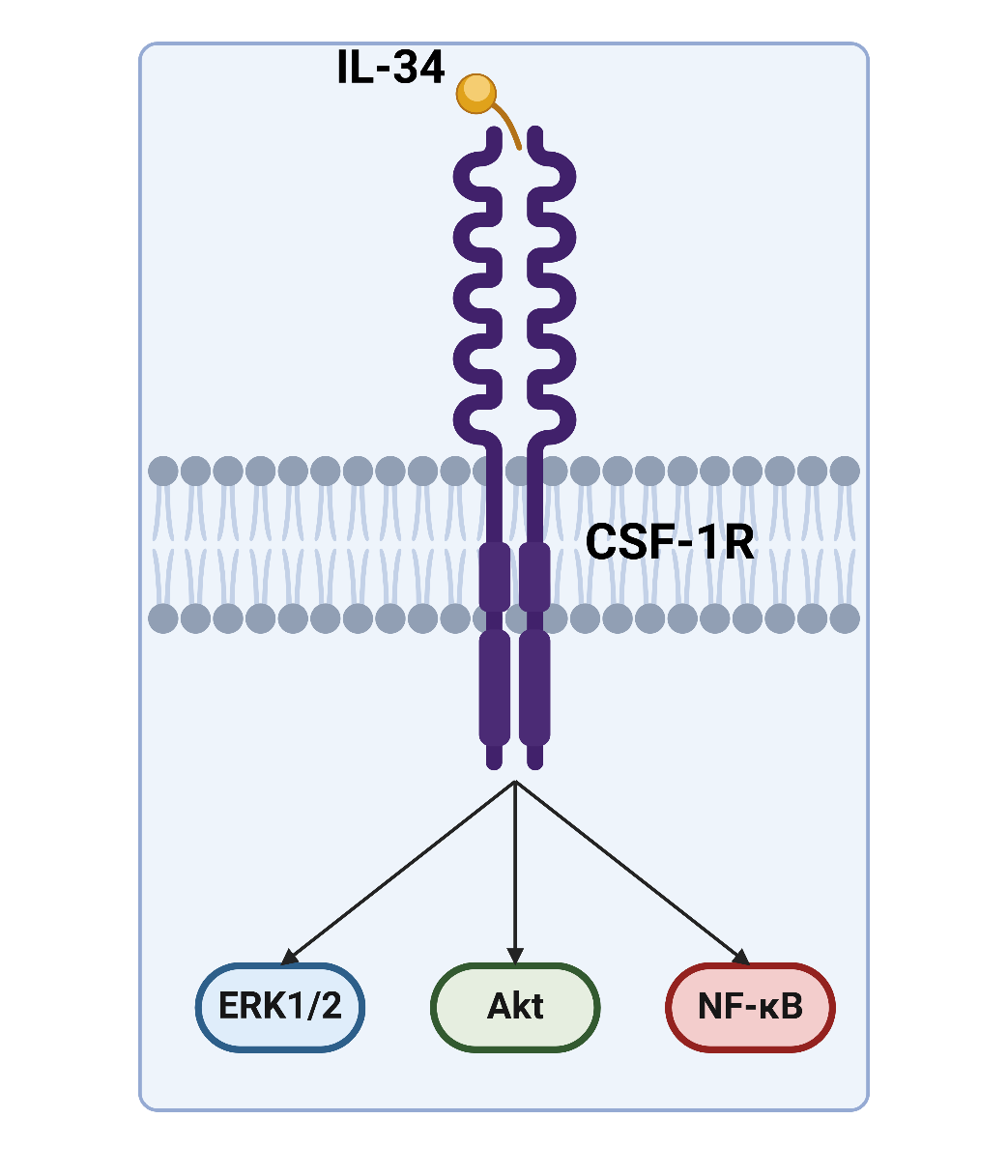
Figure S8*. IL-34 Family:** **IL-34 Binding.** IL-34 binds to the membrane-bound Colony-Stimulating Factor 1 Receptor (CSF-1R). **Intracellular Signaling.** Activation of CSF-1R triggers downstream signaling cascades, such as **ERK1/2:** Associated with diverse cellular processes, such as proliferation, differentiation, and homeostasis; **Akt:** Governs pivotal mechanisms in cell survival and apoptotic regulation; **and NF-κB:** Regulates immune responses and inflammation. This schematic delineates IL-34 binding to CSF-1R, triggering downstream activation of ERK1/2, Akt, and NF-κB signaling cascades. Figure created with BioRender.com.
